# Daptomycin, a last-resort antibiotic, binds ribosomal protein S19 in humans

**DOI:** 10.1186/s12953-017-0124-2

**Published:** 2017-07-01

**Authors:** Michael P. Gotsbacher, Sungmin Cho, Ho Jeong Kwon, Peter Karuso

**Affiliations:** 1 Department of Chemistry and Biomolecular Sciences, Macquarie University, Sydney, NSW 2109 Australia; 2 Department of Biotechnology, Yonsei University, 50 Yonsei-ro, Seodaemun-gu, Seoul, 120-749 South Korea; 3 Present address: School of Medical Sciences (Pharmacology), The University of Sydney, Sydney, NSW 2006 Australia

**Keywords:** Daptomycin, Reverse chemical proteomics, Phage display, DARTS

## Abstract

**Background:**

Daptomycin is a recently introduced, last-resort antibiotic that displays a unique mode of action against Gram-positive bacteria that is not fully understood. Several bacterial targets have been proposed but no human binding partner is known.

**Methods:**

In the present study we tested daptomycin in cell viability and proliferation assays against six human cell lines, describe the synthesis of biotinylated and fluorescently labeled analogues of daptomycin. Biotinylated daptomycin was used as bait to isolate the human binding partner by the application of reverse chemical proteomics using T7 phage display of five human tumor cDNA libraries. The interaction between the rescued protein and daptomycin was validated via siRNA knockdown, DARTS assay and immunocytochemistry.

**Results:**

We have found that daptomycin possesses selective growth inhibition of some cancer cell lines, especially MCF7. The unbiased interrogation of human cDNA libraries, displayed on bacteriophage T7, revealed a single human target of daptomycin; ribosomal protein S19. Using a drug affinity responsive target stability (DARTS) assay *in vitro*, we show that daptomycin stabilizes RPS19 toward pronase. Fluorescently labeled daptomycin stained specific structures in HeLa cells and co-localized with a RPS19 antibody.

**Conclusion:**

This study provides, for the first time, a human protein target of daptomycin and identifies RPS19 as a possible anticancer drug target for the development of new pharmacological applications and research.

**Electronic supplementary material:**

The online version of this article (doi:10.1186/s12953-017-0124-2) contains supplementary material, which is available to authorized users.

## Background

**Scheme 1:**
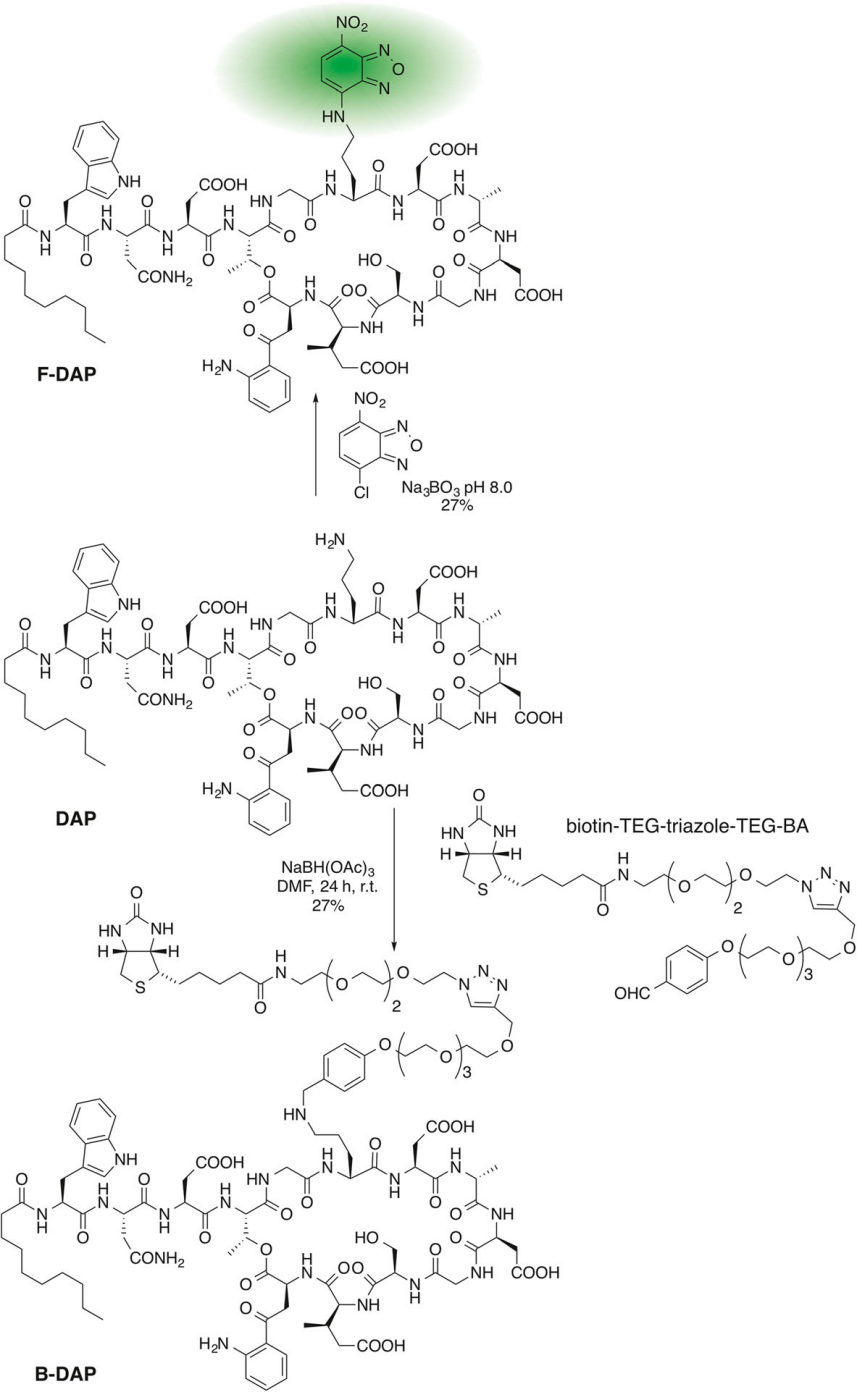
Synthesis of B-DAP and F-DAP

Daptomycin (DAP; Scheme 1) is a natural product (non-ribosomal peptide) from the soil actinobacterium *Streptomyces roseosporus* and comprises a 10 amino acid residue macrolactone (including 3 d-amino acids) with three exocyclic amino acids linked to a fatty acid [1]. DAP has recently been introduced as a last-resort antibiotic with excellent activity against Gram-positive pathogens. It was first approved by the Food and Drug Administration (FDA) for non-topical use in 2003 for the treatment of skin infections caused by Gram-positive bacteria, and in 2006 for non-topical treatment of bacteremia and right-side endocarditis caused by *Staphylococcus aureus*, including MRSA [2]. Despite its clinical importance, the mode of action (MOA) is still unclear, but believed to be a unique mechanism and calcium dependent [3]. Several models have been proposed to explain its antibacterial activity, including: Perturbation of the cell membrane through pore formation, membrane depolarisation or potassium efflux [4–6]; inhibition of the biosynthesis of lipoteichoic acid [7–9]; inhibition of cell wall biosynthesis through the two-component regulatory system YycFG, a membrane-spanning pair of sensor/histidine kinase and response regulator, that is required for viability and functions as a master regulator for cell wall metabolism [10]; and membrane deformation that attracts the conserved cell-division protein DivIVA [11, 12]. Most recently, a comprehensive MOA study indicated that DAP does not form discrete pores or induce membrane deformations, but rather binds to fluid lipid domains in the cell envelope [3].

Very little is known about its interactions of DAP with human cells, with only one reported experiment. In 1990, Canepari exposed human epithelial cells (HEp-2) to radioactively labeled DAP and observed membrane binding to occur in the presence of Ca^2+^ [9]. In their study DAP did not enter the cytoplasm. In addition, bound antibiotic could be removed by washing with EDTA. It was furthermore observed in phospholipid bilayer studies that DAP induces, in a Ca^2+^ dependent manner, substantial lipid flip-flop [5]. This phenomenon is potentially relevant for DAP's entry into human cells, but it is unknown what it interacts with in the lipid bilayer or the cytoplasm.

The side effects of clinical application of DAP (Cubicin®) are generally non-specific, such as nausea, headache, diarrhea and vomiting [13]. However, early Phase I clinical trials showed muscle toxicity at 4 mg/kg every twelve hours [14] and up to 40% of patients develop some muscle toxicity or myalgia [15]. A recent toxicity study on primary rat muscle cell cultures demonstrated that DAP has an effect on the plasma membrane of only differentiated myotubes [16]. As so little is known about the effect of DAP on human cells we initiated a chemical proteomics study to determine the human target(s) of DAP. This is important because it may help elucidate the mode of action underlying the observed side effects but more importantly, it has recently been recognized that bioactive small molecules often possess extensive polypharmacology across target boundaries [17–19]. Therefore, the search for the most avid human protein-binding partner may reveal possible off-label applications of DAP in humans.

Reverse chemical proteomics is the ideal tool to help elucidate targets and off-targets for this complex compound as it allows for rapid discovery of cognate drug-receptor pairs [20]. As so little is known about off-targets or other potential medical applications beyond its antibiotic capacity, we utilized a wide selection of cDNA libraries derived from both normal and diseased cells of various tissues.

## Methods

### Synthesis of probes

B-DAP was synthesized by reductive amination of DAP with a custom made biotinylated linker (Additional file 1: Figure S1-S6). F-DAP was synthesized by an adaptation of the method of Muraih et al. [6] (Additional file 1).

### Reverse chemical proteomics, target identification

#### Reagents and materials

The phage display protocols used were adapted from the Novagen T7 Select Manual [21]. Sodium chloride, potassium chloride, potassium dihydrogen phosphate, Tween-20, IPTG, DNA molecular weight markers and carbenicillin were obtained from Sigma-Aldrich (Castle Hill, Australia). Tryptone, yeast extract, agar and polystyrene Petri dishes were obtained from Bacto Laboratories (Mt. Pritchard, Australia). Glucose, agarose, super-fine resolution agarose and Tris were purchased from AMRESCO (Solon, OH, USA). Acetic acid, glycerol, ammonium chloride, disodium hydrogen phosphate and EDTA disodium salt were obtained from BDH (Darmstadt, Germany). T7Select10-3 human disease cDNA libraries and *E. coli* strain BLT5615 were obtained from Novagen Inc. (Merck; Madison, WI, USA). Nucleotides (dNTPs) were obtained from Bioline (London, UK). Oligonucleotides (primers) were obtained from Sigma-Genosys (Castle Hill, Australia). *Taq*DNA polymerase and QIAquick PCR purification kits were obtained from QIAGEN (Valencia, CA, USA). *Hin*fI restriction endonuclease and NEB buffer 2 were obtained from Promega Corp. (Madison, WI, USA). Electrophoresis grade agarose was obtained from American Bioanalytical (Natick, MA, USA). Nuclease-free water, 1 M magnesium chloride and 20% SDS were obtained from Ambion (Sydney, Australia). Reacti-bind HBC Neutravidin 8-well strip plates (Pierce) were obtained from ThermoFisher Scientific (Scoresby, Australia). Disposable plastic syringes were obtained from Terumo (Tokyo, Japan). Polystyrene 96-well microtiter plates, flexible poly(vinyl chloride) 96-well assay plates and conical 250 mL centrifuge bottles were obtained from Corning (Corning, NY, USA). Reagents and media were prepared according to Table S1 (Additional file 1). Biosafety approval was obtained from the Macquarie University Biosafety Committee (approval number 5201000870).

#### Equipment

Bacterial cultures were incubated in a heated orbital shaker (Thermoline Scientific, Australia). Optical densities were recorded in 1 cm polystyrene semi-micro cuvettes (Sarstedt, Germany) using a BioRad SmartSpec Plus UV spectrophotometer at 600 nm (BioRad, USA). Solutions were centrifuged with a 6 K15 refrigerated centrifuge (Sigma, Germany). DNA was amplified with a C1000 Thermal Cycler (Bio-Rad, USA). DNA sequencing was performed by the Macquarie University DNA Analysis Facility using a *3130xl Genetic Analyzer* (Applied Biosystems, USA). Agarose gel electrophoresis was performed using a Mini-Sub Cell GT system (BioRad, USA) and gels were visualized via Gel-Red stain with a G:Box Chemitransilluminator (ethidium bromide filter) using GeneSnap digital imaging software (SynGene, UK). Water was purified using a Milli-Q Ultrapure Water Purification System (Millipore, USA).

#### Bacterial culturing

Stocks of *E. coli* (strain BLT5615) were stored at –80 °C in 10% glycerol. An initial culture was prepared by streaking a small quantity of this frozen stock onto an LB agar plate and incubating the plate at 37 °C for 16 h and stored at 4 °C for up to 3 weeks. A saturated overnight culture of BLT5615 was prepared by inoculating M9TB (20 mL) with a single bacterial colony from an LB agar plate and then incubating at 37 °C for 16 h with gentle swirling (120–150 rpm). A fresh culture of BLT5615, ready for infection by T7 bacteriophage, was prepared by inoculating M9TB (100 mL) with saturated overnight culture (5 mL) and incubating at 37 °C with vigorous shaking until an OD_600_ of 0.4 was reached (1.5–3 h). IPTG (24%; 100 μL) was added and incubation continued for a further 30 min. The culture was then stored on slushy ice (for up to 24 h) until required.

#### Growth of T7 lysates

IPTG-treated cells (100 mL, BLT5615) were infected with a T7Select cDNA library (1 μL) and incubated at 37 °C with vigorous shaking until lysis had occurred (1–2 h), as indicated by a marked decrease in OD_600_. Immediately following lysis, the lysate was centrifuged at 4700 rpm for 10 min at 4 °C to precipitate cellular debris and the supernatant was decanted into a clean tube containing Tween-20 (1%; 1 mL). The clarified lysate containing 0.01% Tween-20 was stored on slushy ice until required.

#### Stock solutions of biotinylated natural products and controls

Stock solution of B-DAP and B-PROP (1 μmol/mL in DMSO) were stored at –80 °C. Dilutions (1:100) in PBS (pH = 7.4) resulted in 10 nmol/mL solutions and these were stored at –20 °C for up to two days.

#### Biotinylated natural products on neutravidin-coated PS plates

Neutravidin-coated strip wells (Pierce) were pre-incubated with PBS (250 μL) for 1 h at room temperature before use. The wells were emptied and 100 μL probe solutions (10 nM) were applied for 2 h at room temperature. The supernatant was removed, each well washed with PBS (3 × 250 μL) and immediately used for affinity selection.

#### Affinity selections

Clarified T7 phage lysate (200 μL) was added to one well of a neutravidin-coated PS plate that had been derivatized with a biotinylated control compound, and was left to incubate for 1 h at room temperature. The lysate was then transferred to a second well of the plate that had been derivatized with the biotinylated target molecule, and was left to incubate for 3 h at room temperature. The well was washed with PWB (3 × 250 μL) and eluted with SDS (1%; 100 μL) for 30 min at room temperature. Finally, the eluate was diluted with 2xYT (1:10; 900 μL) and stored at 4 °C overnight, during which time a portion of the SDS precipitated. The following day, an aliquot of the eluate (1:10 in 2xYT; 20 μL) was removed, taking care not to disturb any precipitated SDS, and added to fresh IPTG-treated BLT5615 *E. coli* cells (20 mL; OD_600_ 0.4–0.6) for the next round of selection. This procedure was repeated until 7–12 rounds of selection had been completed. The stringency of the washing step was increased with each successive round of selection, from 3 × 250 μL PWB over 10 s in Round 1, to 5 × 250 μL PWB over 2 min in rounds 7–12. PCR of the sublibraries (Additional file 1: Figure S4) was used to monitor convergence.

#### Titring

Standard, round, LB agar plates were pre-warmed to 37 °C. LB agarose (5 mL) was completely melted in a microwave oven and allowed to cool to 50 °C. IPTG-treated BLT5615 cells (250 μL, OD_600_ = 0.8–1) and IPTG (24%; 5 μL) were added to the cooled agarose and the mixture was poured onto one LB agar plate. To allow for the agarose to completely set, the plate was kept uncovered at room temperature for 30–45 min. The phage eluate retained from each round of selection was serially diluted with 2xYT medium from 10^–1^ to 10^–10^ in a flexible 96-well assay plate. A small aliquot (2 μL) of each dilution from each round of selection was dropped onto the surface of the solidified agarose using a multi-channel micropipette (8 × 5 array per plate). The uncovered plate was left to stand at room temperature until the drops had adsorbed entirely into the agarose. Each plate was then incubated for 2–3 h at 37 °C until plaques were clearly visible against the lawn of bacteria. The phage titer was calculated from that particular phage dilution of each round of selection, which contained a countable number (5–50) of plaques. The phage titers for each library from round 2 to round 9 are displayed in Additional file 1: Figure S8.

#### Picking plaques

Serial dilutions (10^1^–10^–7^) with 2xYT were prepared from amplified phage lysate from the final round of selection. LB agarose (5 mL) was completely melted in a microwave oven and allowed to cool to 50 °C. IPTG-treated BLT 5615 cells (250 μL, OD_600_ = 0.8–1), IPTG (24%; 5 μL) and an aliquot (50 μL) of the 10^–7^ dilution were added to the cooled agarose and the mixture was poured onto one LB agar plate. After allowing for the agarose to completely settle, the plate was incubated at 37 °C until plaques were clearly visible against the lawn of bacteria (2–4 h). Individual plaques (24) were collected by stabbing the centre of each plaque with a 10 μL micropipette tip and transferring the tip to IPTG-treated BLT5615 cells (OD_600_ = 0.6–0.8; 100 μL) in a 96-well microtiter plate. The tips were removed and the plate was incubated until complete lysis of bacterial cells occurred in each well (1–2 h). The plate was centrifuged at 4300 rpm for 10 min at 4 °C. An aliquot (40 μL) of the supernatant was transferred into a clean 96-well microtiter plate containing 80% glycerol (10 μL per well) and stored at –80 °C until required.

#### Amplification, sequencing and fingerprinting of cDNA inserts

A solution of phage lysate (0.5 μL) and PCR master mix (19.5 μL, including Taq Polymerase) was prepared and subjected to 20 rounds of thermocycling using the protocol shown in Additional file 1: Table S2. An aliquot of the amplified DNA solution (2 μL) was then incubated with the DNA fingerprinting mix (4 μL) at 37 °C for 1 h.

#### Agarose gel electrophoresis

Electrophoresis-grade agarose (0.6 g) was suspended in 1× TAE buffer (40 mL) and the suspension was boiled in a microwave oven until the agarose had dissolved completely. The 1.5% solution was poured into a casting tray (10 × 7 cm) containing two 15 well combs, and allowed to set for 30–45 min at room temperature. Once the gel had solidified, it was transferred to a gel tank, flooded with 1× TAE, and the combs were removed. For gel electrophoresis of PCR products of single plaques picked from the final round of selection, the agarose concentration was increased to 2%. All digested fingerprinting samples were run in gels made up of super-fine resolution agarose (3%). Each amplified cDNA insert of digested fingerprinting sample (5 μL) was mixed with 6× DNA loading buffer (1 μL) and loaded onto the gel with a micropipette. After all samples had been loaded, the gel was run at 80 V until the bromophenol blue dye had migrated approximately half way down each half of the gel (25–30 min). The gel was then removed from the tank and submerged in a Gel-Red® post-staining solution (3.3×) for 60 min. After de-staining in deionized water (10 min), the gel was visualized using an G:BOX Chemitransilluminator. DNA fingerprinting of random plaques was performed on all cDNA libraries.

#### DNA sequencing

All randomly picked plaques were examined by DNA sequencing. An aliquot of PCR-amplified DNA (10 μL) was purified using a QIAquick PCR purification kit following the manufacturer’s instructions, providing 30 μL solution containing the purified DNA. An aliquot (8 μL) was combined with one PCR primer (1 μM; 4 μL, 4 pmol) and the resulting solution was submitted for DNA sequencing.

### Target validation

#### On-phage binding study

Twelve neutravidin-coated microtiter plate wells were preconditioned with PBS (250 μL) for 1 h at room temperature before use. Six wells were derivatized with the B-PROP and six with B-DAP (100 μL, 10 nM; 2 h). A single phage plaque expressing RPS19 (LiT C1; Additional file 1: Table S3) was reamplified in *E.coli* BLT5615 and the phage lysate clarified (centrifugation) and aliquots (100 μL) incubated in three B-PROP and three B-DAP derivatized wells (2 h, rt). Similarly, wild-type phage (no insert) lysates were incubated in the remaining six wells. The lysates were aspirated and the wells were washed with PWB (10 × 250 μL × 3 s; 4 °C). Any phage particles remaining in the well were eluted with SDS (1%; 100 μL) over 20 min. Serial dilutions were made from the eluates with 2xYT and titered (Fig. 4). Under these conditions, a background of ~10^7^ phage particles is eluted from the strip wells except for the three wells coated with B-DAP and incubated with LiT C1 phage that expressed RPS19. In this case 10^9^ phage were eluted indicating that there is a specific interaction between RPS19 and DAP.

#### Cell culture

MCF7, Huh7, Chang, A549 and HeLa (7–10 passages) cells were cultured in Dulbecco’s modified Eagle’s medium (DMEM, Gibco-BRL, Grand Island, NY, USA). HCT116 (7–10 passages) cells were cultured in Roswell Park Memorial Institute medium (RPMI, Gibco-BRL, Grand Island, NY, USA). U87MG (7–10 passages) cells were cultured in Minimum Essential Medium (MEM, Gibco-BRL, Grand Island, NY, USA). All media contain 10% fetal bovine serum (FBS, Gibco-BRL) and 1% antibiotic-antimycotic (Gibco-BRL). Cells were incubated in a humidified incubator with 5% CO_2_ at 37 °C. Cells were harvested using TrypLE^TM^ Express Enzyme (1 mL; 1 min, Gibco-BRL) and the medium lightly centrifuged 3000 × g, 1 min, 25 °C).

#### Cell proliferation assay

MCF7, HCT116, Huh7, Chang, A549, and U87MG cells (3 × 10^3^) were seeded onto 96-well plates (Tissue Culture Testplate, transparent and flat bottom, SPL Life Science, Pocheon-si, Korea) and maintained for 24 h to stabilize. DAP (0, 5, 10, 20, 40 and 80 μM) was added to each well and incubated for 24, 48 and 72 h. Cell proliferation was measured (in triplicate) using 3-(4,5-dimethylthiazol-2-yl)-2,5-diphenyltetrazolium bromide (MTT; Sigma-Aldrich) at 0.4 mg/mL (final concentration) according to previous report [22].

#### Cell viability assay

MCF7 cells (1 × 10^4^) were seeded onto 24-well plates (Tissue Culture Testplate, transparent and flat bottom, SPL Life Science, Pocheon-si, Korea) and maintained for 24 h to stabilize. DAP (0, 5, 10, 20, 40 and 80 μM) was added to each well and incubated for 72 h. Cell viability was measured (in triplicate) using trypan blue stain (Life technologies, NY, USA).

#### SDS-PAGE and Western blotting

MCF7 cells (6 × 10^4^ cells/well) were seeded onto 12-well plates and grown for 24 h in a humidified incubator with 5% CO_2_ at 37 °C. Cells with trypsin treated were lysed by 2× SDS sample buffer (0.12 M Tris-Cl, pH 6.8, 3.3% SDS, 10% glycerol, 3.1% DTT) and the lysates separated by 12.5 ~ 8% sodium dodecyl sulfate polyacrylamide gel electrophoresis (SDS-PAGE; resolving buffer 1.5 M Tris-Cl, pH 8.8, stacking buffer 0.5 M Tris-Cl, pH 6.8). The running buffer was made of Tris (3 g/L), SDS (1 g/L) and glycine (14.4 g/L). The gels were transferred (2.275 g/L Tris and 7.5 g/L glycine) to PVDF membranes (Millipore, Billerica, MA, USA). Membranes were blocked with 3% skim milk or 1% Bovine Serum Albumin (BSA, Sigma-Aldrich) incubated overnight at 4 °C with the following primary antibodies: anti-RPS19 (sc-100836, Santa Cruz Biotechnology), anti-actin (ab6276, Abcam), anti-HLA A (ab52922, Abcam) and Rabbit and mouse secondary antibody (1:3000 v/v, GE Healthcare, Buckinghamshire, UK) were treated in 3% skim milk or 1% BSA for 1 h at 25 °C. Immunolabeling was detected by an enhanced chemiluminescence (ECL) kit (GE Healthcare) according to the manufacturer’s instructions and detected on a ChemiDoc XRS+ (BioRad, Hercules, CA).

#### siRNA

To knockdown RPS19 mRNA, MCF7 cells (6 × 10^4^ cells/well) were seeded onto 12-well plates in fresh DMEM without FBS. The cells were treated with 40 nM human RPS19 siRNA SMARTpool (L-003771-00, GE Healthcare Dharmacon, Buckinghamshire, UK), 40 nM non-targeting pool (D-001810-10, GE Healthcare Dharmacon) as control and Lipofectamine 2000 transfection reagent (3 μL, Life Technologies, MA, USA). ON-TARGETplus SMARTpool siRNA consisted of 4 siRNAs all targeting human RPS19. ON-TARGETplus Non-targeting Pool siRNA consisted of 4 siRNAs (UGGUUUACAUGUCGACUAA, UGGUUUACAUGUUGUGUGA, UGGUUUACAUGUUUUCUGA, UGGUUUACAUGUUUUCCUA). After 4 h, FBS (10% v/v final) was added to the media. After 24 h, the efficiency of siRNA knockdown was analyzed by Western blotting.

#### DARTS

MCF7 cells (6 × 10^6^) were lysed by homogenising in PBS buffer treated with a protease/phosphatase inhibitor cocktail (1/2 tablet/25 mL; Pierce, Rockford. IL, USA) and the protein concentration was measured using a Bradford assay. The cell lysate (protein concentration; 1.5 mg/mL, 100 μL) was aliquoted into 1.5 mL tubes. The cell lysate was incubated at 4 °C with 0 or 100 μM DAP (in 3 μL DMSO) for 4 h with rotation and then pronase (in water) added (0.1 mg/mL final concentration) and incubated for 0, 5, 10 and 20 min at 25 °C. Protease activity was stopped by addition of 6× SDS sample buffer to a final concentration of 1× SDS and the samples boiled for 7 min. The protein levels of RPS19 and HLA-A were quantified by using Western blotting (see above).

#### Immunocytochemistry

For competition assay of DAP to F-DAP, HeLa cells (1.5 × 10^5^ cells/well) were seeded in 6 well plates with coverslips and incubated at 37 °C for 24 h. DAP (0 or 50 μM final concentration), Biotin (50 μM), and Cryptopleurine (50 μM) were added and incubated for 1 h followed by F-DAP (20 μM final concentration) for an additional 30 min. Cells were washed (3 × DMEM) and fixed with 4% paraformaldehyde in PBS for 5 min. Cells were analyzed by confocal microscopy (LSM700, Carl Zeiss, Oberkochen, Germany). Excitation at 405, and 488 nm was used for Hoechst 33342, and F-DAP respectively with 435, and 518 nm emission filters for Hoechst, and F-DAP, respectively.

### Statistical analysis

Linear and non-linear least squares regression analysis was performed using Prism 5.0 (GraphPad Software, USA). All quantitative results are expressed as mean ± standard error (S.E.M) and the Student’s *t*-test (GraphPad Prism) was used to determine statistical significance between two groups. A *p*-value < 0.05 was considered statistically significant (* *p* < 0.05, ** *p* < 0.01). One-way ANOVA test (GraphPad Prism) was used to determine statistical significance for a series.

### Molecular modeling

The high resolution (3.6 Å) cyroEM structure of the human ribosome (PDB ID 5T2C) [23] was loaded into MOE2016.08 (Chemical Computing Group). RPS19 was extracted from the structure and prepared for docking by 3D protonation (pH 7.2, 300 K, 0.1 M salt with a dielectric of 80) and the structure relaxed, tethering all heavy atoms and allowing the hydrogens to move during minimization (RMS < 0.001). A database of the NMR solution structures of DAP (PDB ID 1XT7, 1T5M and 1T5N) [5, 24] was constructed and docked (default conditions) against the isolated RPS19. Docked structures were ranked according to their final interaction energy calculated to be the sum of the van der Waals electrostatics and solvation energies, under the Generalized Born solvation model (GB/VI).

## Results

### Biological activity of DAP in human cell lines

**Fig. 1:**
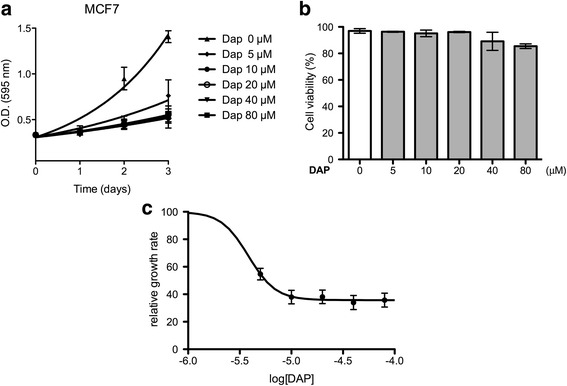
The proliferation of MCF7 (breast cancer cells) was inhibited by DAP without cell toxicity. **a** Effect of DAP on the proliferation of MCF7 cells was measured up to 3 days. DAP was treated in triplicate at each concentration. **b** Effect of DAP on the viability of MCF7 cells was investigated by using a trypan *blue* staining assay (*p* = 0.003, one-way ANOVA). **c** The growth rates from (**a**) (t = 3 d) plotted against log[DAP]. 100% equal the growth rate of untreated cells

DAP was bioassayed against six human cell lines, up to 80 μM for 3 days (Table 1; Additional file 1: Figure S9). No toxicity, even at the highest concentration, was observed, but there was growth inhibition of MCF7 and HCT116 cell lines. MCF7 growth rates were strongly affected even at 5 μM (Fig. 1a and c). Cell viability after 3 days was confirmed using trypan blue (Fig. 1b) and the growth rate (percentage growth compared to no treatment) supported growth inhibition and not lethality. The growth inhibition for MCF7 cells was confirmed using trypan blue (Fig. 1b).

**Table 1:** The effect of DAP on the proliferation of various cell lines. The normal and cancer cells were treated with DAP up to 80 μM. The effect of DAP on cell proliferation was measured for three days with MTT assay after drug treatment. GI_50_ values were calculated on sigmoidal dose-response graphs after three days by GraphPad Prism

	Normal cells	Cancer cells				
Cell lines	Chang	MCF7	A549	HCT116	U87MG	Huh7
GI_50_ (μM)	>100	3.8	>100	6.5	>100	>100

To identify the human binding partner(s) for DAP, we applied reverse chemical proteomics, using biotinylated DAP (B-DAP) as the bait and colon, liver, lung and breast tumor cDNA libraries displayed on T7-bacteriophage.

### Chemistry

DAP was conjugated to a biotinylated linker, biotin-TEG-triazole-TEG-BA, (Scheme 1) via a reductive amination [25]. The linker was constructed from Biotin-NHS and specifically designed tetraethyleneglycol linkers (Additional file 1). A control probe (B-PRO) was synthesized using propylamine instead of DAP, with exactly the sample biotinylated linker to mimic the side chain ornithine (Additional file 1). The structure of the probes was confirmed by NMR spectroscopy and mass spectrometry (Additional file 1). Antimicrobial assay of B-DAP confirmed that the probe was still active as an antibiotic albeit, 10× less active than DAP (Additional file 1: Figure S7). Mass spectrometry revealed that B-DAP contained ~60% B-DAP with biotin sulfoxide. It is likely that the copper catalyzed click reaction used in constructing biotin-TEG-triazole-TEG-BA resulted in partial oxidation of biotin. However, while several oxidative side-reactions are known for this reaction [26], this is the first example of oxidation of a sulfide. The separation of those two species proved unnecessary because biotin sulfoxide also binds to neutravidin, but with a lower affinity [27], and the presence of the sulfoxide cannot affect the biopanning. In addition, use of excess probe would wash away the lower affinity biotin sulfoxide probe.

**Fig. 2:**
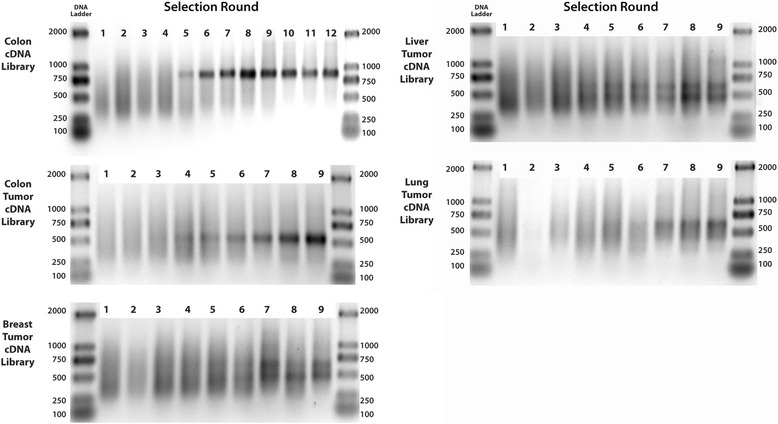
Agarose gel electrophoresis of phage DNA inserts amplified by PCR from cDNA libraries (colon, colon tumor, breast tumor, liver tumor and lung tumor) after 9–12 rounds of biopanning against B-DAP immobilized on a neutravidin-coated microtiter strip wells

Strip wells coated with neutravidin (Pierce) were incubated with excess B-DAP or B-PRO (2 h), rinsed and the B-PRO derivatized wells incubated with the lysate from five T7 phage-displayed human cDNA libraries (normal colon, colon cancer, breast cancer, liver cancer and lung cancer) for 1 h and then the lysate transferred to B-DAP derivatized wells (3 h). The lysate was aspirated and the wells washed with buffer and the adherent phages eluted with SDS. Reapplication of the eluted phages (*E. coli*) produced round 1 sublibrary, which was preincubated with B-PRO coated wells and then B-DAP well, washed and eluted to produce round 2 sublibrary. This process was repeated 9–12 times to isolate the most avid binding phages from the initial cDNA libraries (Fig. 2).

**Fig. 3:**
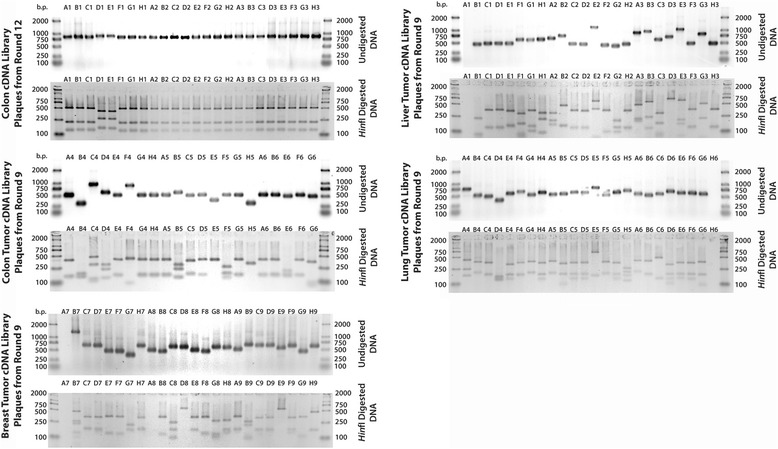
Agarose gel electrophoresis of PCR products obtained from normal colon, breast tumor, colon tumor, liver tumor and lung tumor individual plaques after nine rounds of selection with B-DAP immobilized on a neutravidin-coated plate. The DNA inserts, which were amplified using generic T7 primers, were also digested with *Hin*fI to produce unique DNA fingerprints of each clone. Clones that appeared more than once were sequenced

The selections of random plaques from the final round of biopanning were subject to PCR amplification and *Hin*fI fingerprinting, and separated by gel electrophoresis (Fig. 3). The *Hin*fI digestion of the PCR products allowed fingerprinting of the rescued clones and those that appeared several times were purified and sequenced (Table 2).

**Table 2:** DNA sequencing of PCR products obtained from individual plaques after nine rounds of selection with B-DAP immobilized on a neutravidin-coated PS microtiter plate. The DNA sequences of each plaque are available upon request

Library	Plaques	Gene Identified from DNA Sequence	Notes	Frame
Col^a^	A3	leukocyte receptor cluster (LRC) member 8	minor fraction of CDS	1
Col^a^	D1	tenascin C (TNC)	500 bp of 6400 bp CDS	1
CoT	A4, E4, G4, H4	ribosomal protein S19	350 bp of 420 bp CDS	1
CoT	B4	dynamin 2 (DNM2)	only late 180 bp of 2500 bp CDS	2
CoT	B5	sphingomyelin phosphodiesterase 4	outside CDS	-
LiT	C1, D1, C2, H3	ribosomal protein S19	350 bp of 420 bp CDS	1
LiT	E1	ribosomal protein S19	360 bp of 420 bp CDS	1
LiT	B2	required for meiotic nuclear division 5 homolog B	gene in backwards	-
LiT	A3	UDP-Gal:betaGlcNAc beta 1,4- galactosyltransferase, polypeptide 5	gene in backwards	-
LiT	B3	RAD21 homolog (S. pombe)	500 bp of 1800 bp CDS	1
LuT	A4, C5	ribosomal protein S10	last 360 bp of 500 bp CDS	1
LuT	C4	ribosomal protein S19	300 bp of 420 bp CDS	1
BrT	B7	v-erb-b2 erythroblastic leukemia viral oncogene homolog 2, neuro	very late 480 bp of 3680 bp CDS	1
BrT	D7	ribosomal protein S19	300 bp of 420 bp CDS	1
BrT	A9	ribosomal protein S19	330 bp of 420 bp CDS	1
BrT	D9	ribosomal protein S10	430 bp of 500 bp CDS	1
BrT	E9	BAC clone RP11-22 K23	outside CDS	-
BrT	H9	LYR motif containing 5	230 bp of 270 bp CDS	1

^a^ Plaques derived from this library were picked after 12 rounds of selection

The majority of clones that were in frame with the T7 bacteriophage coat protein, displayed ribosomal protein S19 (RPS19) on their surface.

### Target validation

**Fig. 4:**
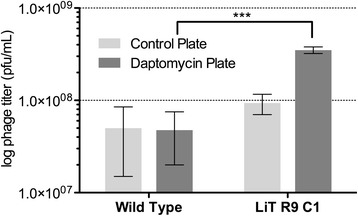
On-phage binding study comparing the affinity of the RPS19-displaying phage clone C1 (from liver tumor sublibrary of round-9) for neutravidin-coated plates derivatized with a control compound and a similar plate derivatized with B-DAP

Initially, an on-phage binding assay was performed to determine if the T7 phage displaying RPS19 clones (clone C1 from the liver tumor cDNA library) had greater affinity for neutravidin-coated plates derivatized with B-DAP than for similar plates derivatized the control (B-PRO). A T7 clone with no cDNA insert, was used as a negative control. The assay was conducted in triplicate and the results show a statistically significant (*p* < 0.001, one-way ANOVA) higher binding to DAP derivatized plates (Fig. 4).

**Fig. 5:**
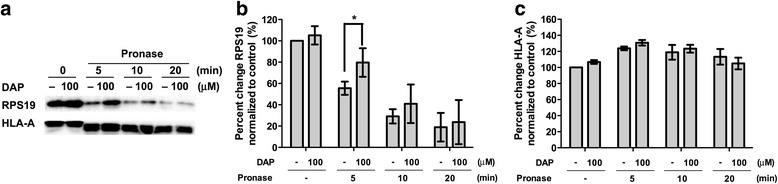
Validation of the binding of DAP to RPS19 *in vitro* and in vivo. **a** Western blotting of DARTS analysis in respect with RPS19 and HLA-A (loading control) in DAP and pronase treatment. **b** graphical representation of **a** for RPS19 run in triplicate. * designates *p* < 0.05. **c** graphical representation of **a**) for HLA-A run in duplicate

**Fig. 6:**
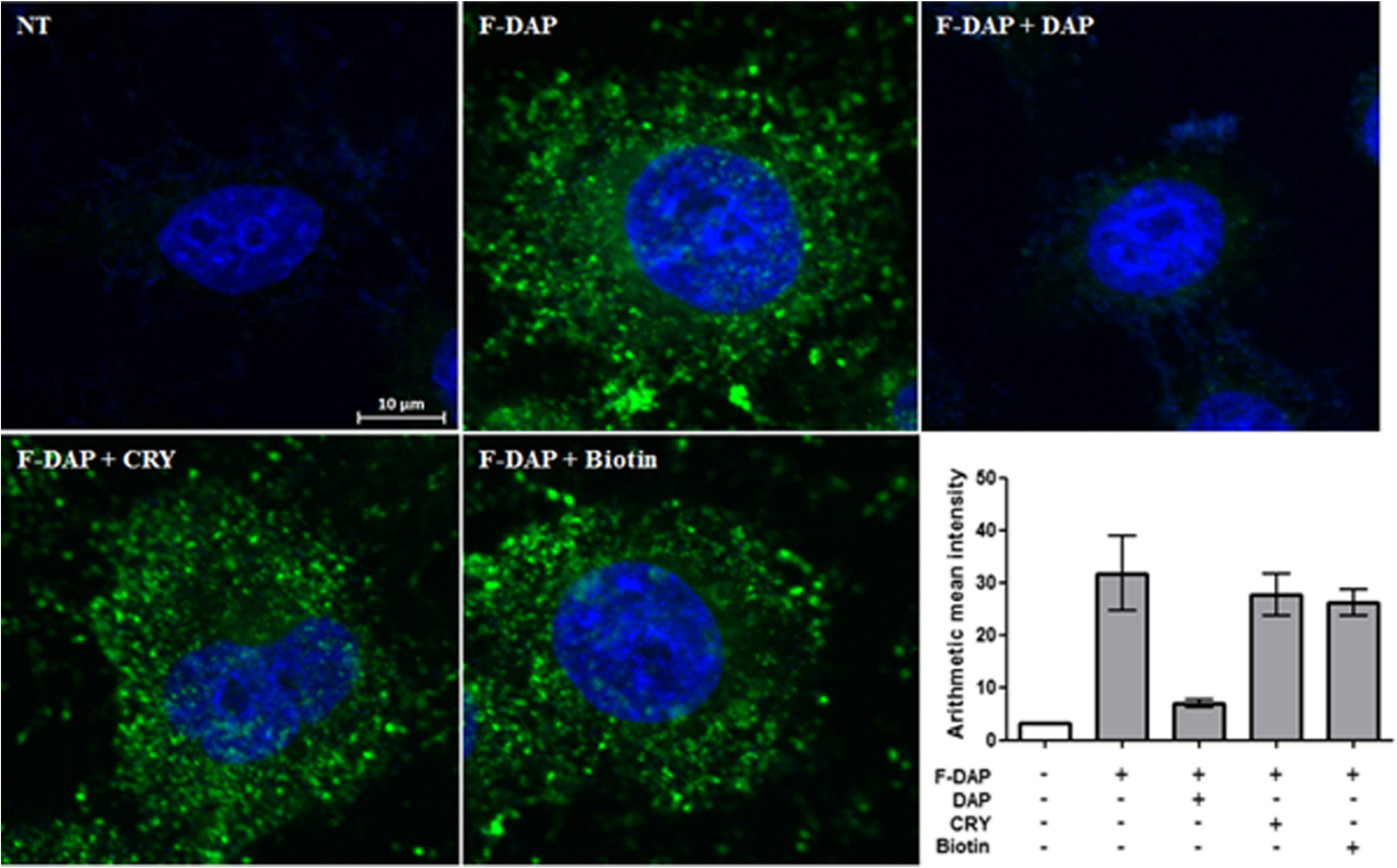
In vivo competition assay between DAP and F-DAP in HeLa cells. Hoechst 33342 (*blue*), F-DAP (*green*). The graph shows mean fluorescence intensity of F-DAP

**Fig. 7:**
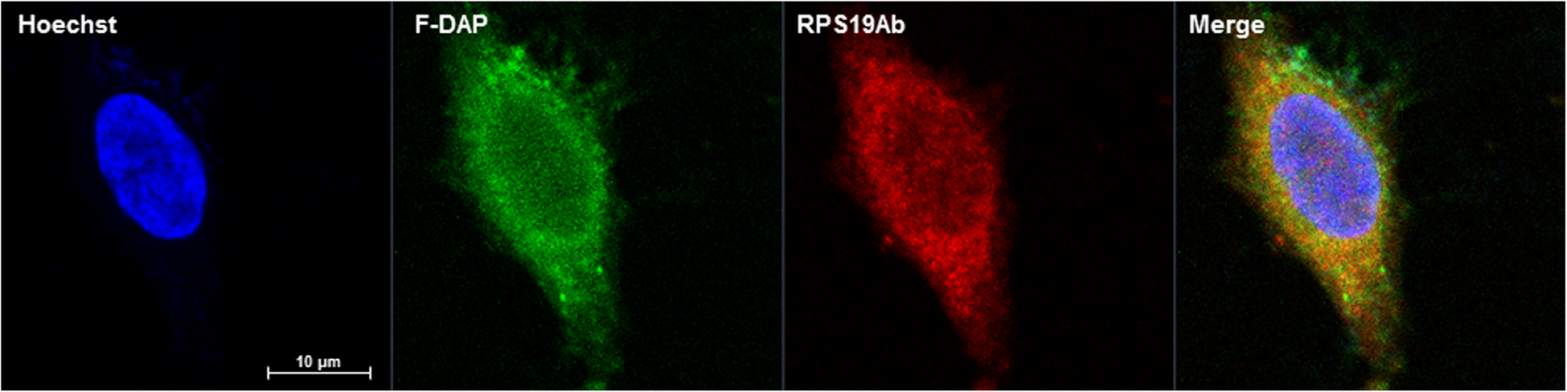
Confocal images of HeLa cells. Hoechst 33342 (*blue*), F-DAP (*green*) and RPS19Ab (*red*), colocalization (*orange*)

Drug affinity responsive target stability (DARTS) [28] was used to validate the direct binding of DAP to RPS19 *in vitro*. With 0.1 mg/mL of pronase for 5 min there was a significant (*p* = 0.0478, Students *t*-test) protection from hydrolysis of RPS19 in MCF7 whole cell lysate in the presence of 100 μM DAP (Fig. 5). In contrast HLA-A was degraded constantly irrespective of the presence of DAP. The binding of DAP to RPS19 was further investigated in HeLa cells through confocal microscopy. In order to visually confirm the binding of DAP to RPS19, F-DAP was synthesized and used in a competitive binding study. Cells that were pretreated with unlabeled DAP and then stained with F-DAP (Fig. 6, panel 3), showing little or no staining. In contrast, pretreatment of cryptopleurine (CRY) and biotin did not compete with F-DAP staining, demonstrating that F-DAP and unlabeled DAP share the same binding protein and site of the protein in the cytosol of HeLa cells (Fig. 6). F-DAP and RPS19Ab were also found to partially colocalize in the cytosol of HeLa cells where RPS19 is mainly localized (Fig. 7), suggesting that F-DAP and DAP bind to RPS19 in living cells.

**Fig. 8:**
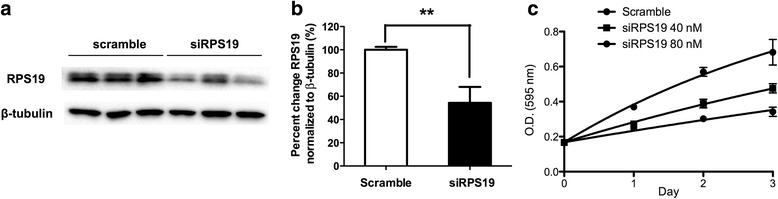
The effect of RPS19 knock down on the proliferation of MCF7 cells. **a** Western blot of MCF7 cells treated with 40 nM small interfering RNA against RPS19 (siRPS19). β-Actin was used as a loading control and scrambled small interfering RNA (Scramble) was used as a negative control. **b** Quantification of **a** is shown in the bar graph run in triplicate (*p* < 0.01; Students *t*-test). **c** Effect of RPS19 on the proliferation of MCF7 cells was measured up to 3 days after siRNA treatment in triplicate

The effect of knocking down RPS19 on the proliferation of MCF7 cells was investigated using siRNA (Fig. 8a-c). Treatment of MCF7 cells with 40 nM siRNA against RPS19 (siRPS19) or random siRNA (Fig. 8a), resulted in a 50% reduction in RPS19 levels (*p* = 0.024) compared to control (β-actin; Fig. 8b). At 80 nM siRPS19 we observed 72% growth inhibition of MCF7 cells (Fig. 8c). This dose dependent effect on growth reduction is similar to what was observed for DAP (Additional file 1: Figure S9).

Docking of DAP to the isolated structure of human RPS19, taken from the recently released high resolution cyroEM structure of the human ribosome [23] resulted in 15 lowest energy docked structures. Four of the five lowest energy docked structures are to the same site on RPS19, at the interface between rRNA and RPS19 (Fig. 10). In these structures, several of the carboxy groups of DAP took up positions close to the phosphate residues in the rRNA that RPS19 naturally binds too.

## Discussion

This is the first report of the effect of DAP against human cells and indicates that there might be a specific target for DAP in humans that could explain the observed side effects or point the way to off-label applications of DAP in human pharmacology. Bioassay of DAP against a panel of human cell lines surprisingly revealed selective growth inhibition against breast (MCF7) and colon (HCT116) cancer cell lines. It appears that the differences in RPS19 expression levels between cell lines could be one of the reasons why MCF7 and HCT116 were more sensitive to DAP. Further assessment on the expression level of RPS19 in various cell lines will be conducted in a follow-up study.

**Fig. 9:**
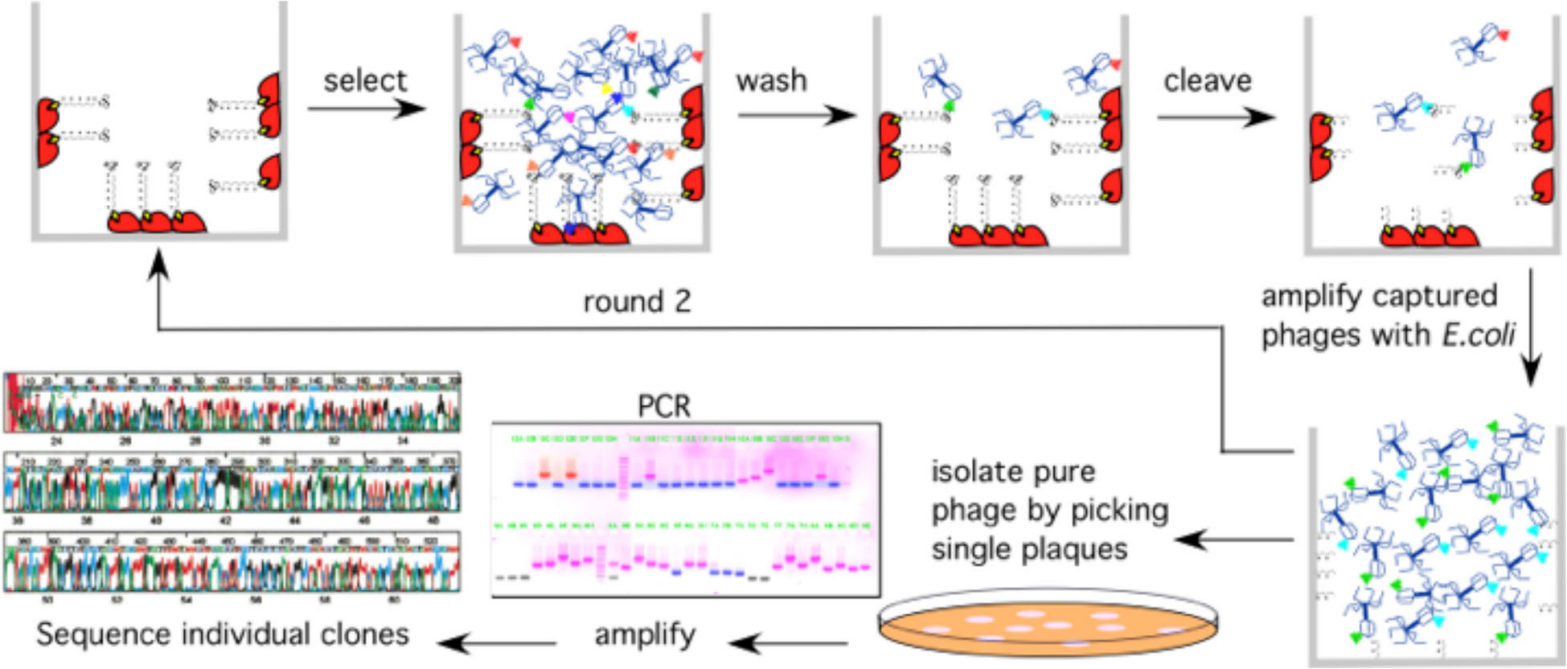
The biopanning process begins with biotinylation and immobilization of a small molecule onto a surface coated with neutravidin (*red*). Introduction of a DNA library displaying a small number (1–15) of copies of the encoded protein, is introduced into the well and, non-binding phages removed by washing. Bound phages are eluted and amplified (*E. coli* BLT5615) and the process repeated until the library converges on the most avid binding target protein(s). Individual phage plaques are sequenced to determine the identity of the displayed protein

There are many methods to link small molecules with their targets that all have specific advantages and disadvantages [20, 29, 30]. One of the most attractive methods (reverse chemical proteomics) is a relatively unbiased, genome wide methodology that starts with the transcriptome, which is cloned into an amplifiable vector that then displays the entire proteome of the original cell. Iterative biopanning (Fig. 9) can then be used to isolate the most avid binding partners of a bait molecule, regardless of how dilute the original mRNA was. Here we have chosen the underutilized T7-bacteriophage system, which has proven useful in the past for the isolation of small molecule binding proteins [31–36].

For DAP, several studies have shown which sites are suitable for derivatization without significant loss of activity and which must not be modified. From SAR studies on Orn6, it became clear that the γ-amino group is not essential for whole cell activity, but derivatization with various functional groups impacted the antibiotic activity [25]. The authors determined that the amine is required for activity but that this does not need to be a primary amine. Consequently, biotinylation was achieved through arylation of the ornithine via a reductive amination. Antibiotic activity was retained, albeit at a slightly lower level (EC_50_ = 41 μM for B-DAP *c.f.* 5.4 μM for DAP against *S. aureus*; Additional file 1: Figure S7). As *N*-alkylation of DAP is known to have no effect or enhance antibiotic activity, the observed reduction in activity is most likely due to bioavailability, with the long PEG linker and biotin hindering cell permeability.

DAP is susceptible to both alkaline and acidic degradation, giving rise to three major degradation products [37]. Under strongly alkaline conditions, ester hydrolysis between Thr4 and Kyn13 results in a ring opened product. In mildly acidic conditions (pH = 3–6) a two-step pathway results in the succinimido intermediate (“anhydro-DAP”) at Asp9 and subsequent reversible formation of two aspartic acid isomers. At lower pH, other degradation pathways occur. B-DAP was shown stable under the biopanning conditions (pH 7.4 for up to 6 h) for at least 12 h (Additional file 1: Figure S6).

After 9–12 rounds of biopanning of B-DAP against several cancer cDNA libraries, and one normal colon library, all pathological libraries started to converge onto dominant clones (Fig. 2). Analysis of the DNA sequences from a subset of the rescued phages (Fig. 3) clearly showed that the clones displaying the ribosomal protein S19 (RPS19) were the most abundant in most libraries. They comprised 11 out of 23 random plaques selected from the colon tumor library, 8 out of 23 from the liver tumor library, 7 out of 23 from the lung tumor library, and 5 out of 23 from the breast tumor library (Table 2). In conjunction with an exponential rise in titer (Additional file 1: Figure S8), this indicates a successful selection.

**Fig. 10:**
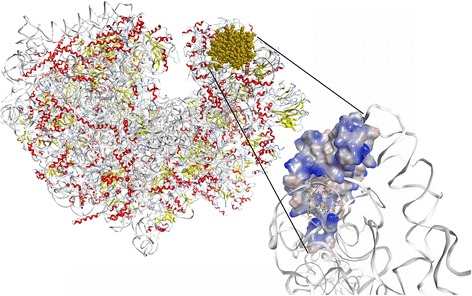
CryoEM structure of the human ribosome (image of PDB ID 5T2C [36]) rendered in MOE2016. RPS19 is shown in *gold*. The inset shows the lowest energy DAP conformation docked to free RPS19. The solvent accessible surface is colored by hydrophocity (*blue is hydrophobic*, *pink is hydrophilic*). rRNA is indicated as a *white ribbon* shows that DAP can bind to the RPS19-rRNA interface

Alignment of all converted RPS19 proteins with the authentic human RPS19 (Additional file 1: Table S3) revealed that all the rescued clones were missing the first 21–39 amino acids with various lengths of 3'-UTR, suggesting that the binding of DAP to RPS19 did not require the first 39 amino acids. Recently the high resolution total structure of the human ribosome has been published and shows RPS19 to be located on the head of the 40S subunit, extending well into the functional center of the 40S subunit [23]. The protein is exposed (Fig. 10) and the binding of DAP could interfere with protein synthesis.

The RPS19 protein is a component of the 40S ribosomal subunit and belongs to a family of ribosomal proteins restricted to eukaryotes (and archaea). There is no homologue of RPS19 in bacteria. It is essential for yeast viability and for early stages of development in mice [38, 39]. Disruption as well as point mutations of the RPS19 gene in yeast and human cells affect maturation of the pre-ribosomal RNA (pre-rRNA) and blocks the production of the 40S ribosomal subunits [38, 40, 41]. Knocking down RPS19 with siRNA leads to dramatic growth inhibition (Fig. 8), but not death of MCF7 cells in culture. Mutations of RPS19, as well as of two other ribosomal proteins, RPS24 and RPS17, have been linked to the rare congenital disease Diamond-Blackfan Anemia [41–45]. RPS19 may also have extra-ribosomal functions. For example, Kondoh et al. [46] reported higher expression levels of RPS19 in certain colon cancer cell lines, compared to normal colon tissue, which increased concomitantly with tumor progression.

From an “on-phage” binding assay, it was clear that phage expressing RPS19 had a higher affinity for B-DAP derivatized surfaces than wild-type phage (Fig. 4). However, the DARTS assay has been proven to be a much more efficient and reliable method to validate the interaction of a small molecule with its target protein *in vitro* [28, 29, 47]. The DARTS assay on MCF7 cell lysate showed that RPS19 is more resistant to hydrolysis in the presence of DAP, suggesting that DAP binds directly to RPS19 (Fig. 5a, b). HLA-A, an MHC class I protein, was used as an internal control. The band for HLA-A (Fig. 5a) was down-shifted, indicating a facile cleavage site in the protein but the remaining fragment was quite resistant to pronase (Fig. 5c).

Staining of fixed HeLa cells with F-DAP and RPS19Ab (Fig. 6) showed a general diffuse staining of the cytoplasm that colocalized by 80%. However, antibody staining requires permeabilization of the cells with detergent, which was found to disrupt F-DAP staining. Much clearer staining is obtained with F-DAP alone (Fig. 6), which was found to be freely cell permeable and stained structures inside the cells. Small molecule specific staining was confirmed by using DAP pretreatment, which effectively prevented F-DAP binding. As controls, cryptopleurine (CRY), which is known to bind the 40S subunit of the ribosome [48], did not disrupt cellular staining by F-DAP. The observed staining is consistent with ribosomes in HeLa cells [49].

In the presence of a mixture of siRNAs against RPS19, cell proliferation was suppressed. It can be inferred the binding of DAP to RPS19 and knocking down RPS19 have the same general outcome on cancer cell proliferation. Molecular modelling (Fig. 10) suggested that DAP could bind to the rRNA interface with RPS19. In so doing it might interfere with incorporation of RPS19 into the ribosome and could explain why knocking down RPS19 and DAP treatment had the same qualitative outcome for cancer cells. Taken together, these data suggest that DAP could be inhibiting the cellular function of RPS19 and that RPS19 is a viable drug target for further drug development.

## Conclusion

In conclusion, a genome wide, unbiased reverse chemical proteomics screen of DAP binding protein(s) in several human cancer cells identified RPS19 as a biophysically and biologically relevant target protein of DAP in humans. The interaction of DAP with RPS19 was validated using an on-phage binding assay, a label-free DARTS assay and colocalization of F-DAP with RPS19Ab. This is the first report that DAP may have selective anticancer activity and its associated human target, RPS19, could be a promising drug target protein. Further studies on the exact binding site of DAP and of the anticancer activity of DAP are warranted and may allow off-label applications of this last resort antibiotic in the field of cancer therapeutics.

## Additional file

Additional file 1: Supplementary data (synthetic routes, ^1^H NMR and MS/MS spectra of reported compounds, supplementary figures and tables) associated with this article can be found, in the online version, at http://[…]. (PDF 2959 kb)

## Abbreviations

BA: benzylaldehyde
B-DAP: biotinylated daptomycin
B-PROP: biotinylated propylamine
CRY: cryptopleurine
DAP: daptomycin
DARTS: drug affinity responsive target stability
F-DAP: fluorescent daptomycin
RPS19: ribosomal protein S19
RPS19Ab: Antibody against ribosomal protein S19
siRPS19: siRNA against RPS19
TEG: triethyleneglycol
