# Supplementary material for: Daptomycin, a last-resort antibiotic, binds ribosomal protein S19 in humans
Source: Proteome Sci. 2017 Jul 1;15:16. doi: 10.1186/s12953-017-0124-2 (PMC5494143; doi:10.1186/s12953-017-0124-2)
Supplement: Additional file 1: — Supplementary data (synthetic routes, 1H NMR and MS/MS spectra of reported compounds, supplementary figures and tables) associated with this article can be found, in the online version, at http://[…]. (PDF 2959 kb) [file 12953_2017_124_MOESM1_ESM.pdf]

Supplementary Information:

Experimental Section

1. Synthesis of Probes

General. Unless otherwise stated, all chemicals and reagents were received from Sigma-Aldrich (Castle Hill, Australia) and used without further purification. Chloroform, dichloromethane, ethyl acetate, methanol and tetrahydrofuran were obtained from ChemSupply (Gilman, Australia), and were glass distilled before use. NBD-Cl (4-chloro,7-nitro-2,1,3-benzoxadiazol was obtained from Fluka (Fluka, St. Louis, MO, USA). The following solvents were distilled prior to use: ethyl acetate from anhydrous potassium carbonate, THF under nitrogen from sodium/potassium with benzophenone as indicator. HPLC grade acetonitrile was obtained from BDH/Merck (Darmstadt, Germany), and was used without further purification. Daptomycin was purchased from OChem Incorporation (Des Plaines, IL, USA) and used without any further purification.

All reactions were performed under an inert atmosphere of dry N_2 in oven-dried (80 °C) glassware. ^{1}H and ^{13}C NMR spectra were recorded on a Bruker Avance DPX-400 400 MHz or AVII-600K 600 MHz NMR spectrometers. Proton chemical shifts are reported in parts per million from an internal standard of residual DMSO (2.50 ppm) or chloroform (7.26 ppm), and carbon chemical shifts are reported using an internal standard of residual DMSO (39.52 ppm) or chloroform (77.16 ppm). Proton chemical data are reported as follows: chemical shift, multiplicity (s, singlet; d, doublet; t, triplet; m, multiplet; br, broad), coupling constant, assignment. Low resolution mass spectrometry (MS) was performed by electrospray ionisation (ESI) MS in positive polarity mode on a Shimadzu LC-20A prominence system coupled to a LCMS-2010 EV mass spectrometer using LCMSsolution 3.21 software. LC-MS experiments were carried out on a Gemini C18 column (Phenomenex, Australia) 150.0 × 2.00 mm, 110Å, 3 µm. High resolution mass data were obtained from ESI in positive polarity mode on a Bruker 7T Fourier Transform Ion Cyclotron Resonance (FTICR) mass spectrometer, performed by the Mass Spectrometry Unit at University of Sydney, Australia. MS/MS experiments were performed on a HCT 3-D ion trap (Bruker Daltonics) running DataAnalysis 4.0 (Bruker) in the Australian Proteome Analysis Facility, Sydney. Full scan (*m/z* 200-2200) was followed by a data-dependent fragmentation of the two most abundant signals using alternating collision induced dissociation (CID) and electron transfer dissociation (ETD). HPLC analysis was performed on a Shimadzu 10AD-VP system running Class-VP 7.4 SP1 software. Analytical, semipreparative and preparative HPLC were performed on Gemini C18 HPLC columns (Phenomenex, Sydney, Australia): Gemini-NX C18 250.0 × 4.6 mm, 110Å, 5 µm; Gemini C18 250.0 × 10.0 mm, 110Å, 10 µm; Gemini-NX C18 150.0 × 21.2 mm, 110Å, 5 µm. TLC was performed with Merck Kieselgel 60 F_{254} plates with viewing under ultraviolet light (254 nm and 365 nm) and/or by heating after treatment with appropriate TLC staining solutions. Flash column chromatography was performed on silica gel (60 Å 0.040–0.063 mm, 230-400 mesh ASTM from Merck).

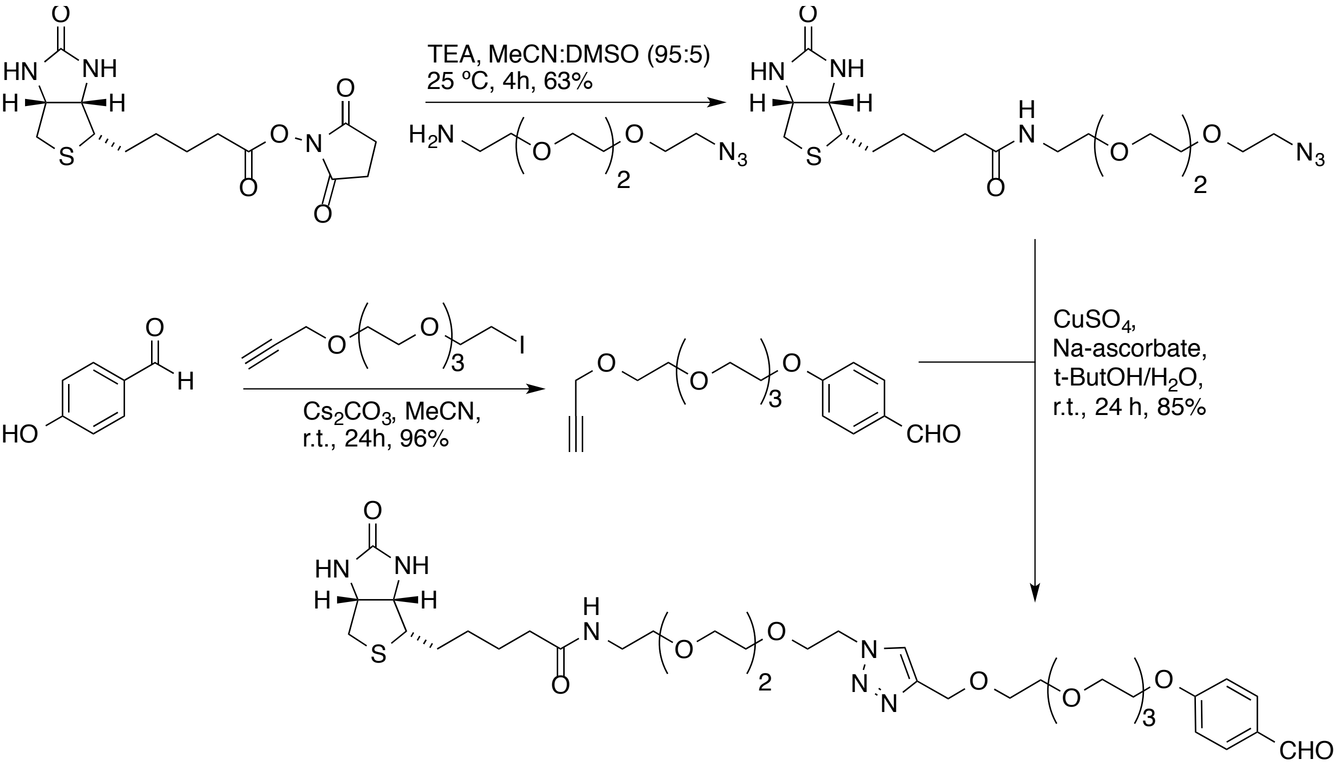

Scheme S1. Synthetic route for biotinylated polyethyleneglycol (PEG) based linker. Starting from biotin and tetraethyleneglycol (TEG), a biotin-TEG-triazole-TEG-benzaldehyde linker was synthesized, which was suitable for reductive amination with the primary amines of daptomycin or propylamine. For synthetic route of **B-DAP** and **B-PROP** see main manuscript.

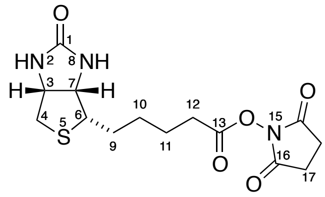

Biotin N-hydroxysuccinimide ester. A solution of biotin (200 mg, 0.80 mmol), NHS (92 mg, 0.80 mmol) and DCC (168 mg, 0.80 mmol) in THF:DMSO (95:5; 20 mL) was stirred at room temperature for 18 h. The THF was then removed under a stream of nitrogen and the DMSO by lyophilization. The residue was recrystallized from isopropanol, yielding biotin *N*-hydroxysuccinimide ester (biotin-NHS ester) as a white powder (235 mg, 88%), m.p. 201-202 °C (Lit. [1] 200-202 °C). ^{1}H-NMR (400 MHz, DMSO-d_6) δ 6.41 (s, H-8), 6.35 (s, H-2), 4.29 (m, H-3), 4.14 (m, H-7), 3.10 (m, H-6), 2.82 (dd, J = 12.8 Hz, 5.0 Hz, H-4β), 2.80 (s, $(H\text{-}16)_4$), 2.66 (t, J = 7.3 Hz, $(H\text{-}12)_2$), 2.57 (d, J = 11.4 Hz, H-4α), 1.40–1.68 (m, H-9 – $(H\text{-}11)_2$). Mass spectrum (ESI+) *m/z:* 364 ($[M + Na]^+$), 342 ($[M + H]^+$).

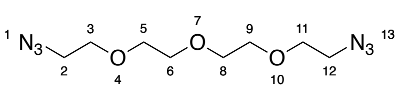

TEG-diazide (1-azido-2-(2-(2-(2-azidoethoxy)ethoxy)ethoxy) ethane). TEA (27.3 mL, 195.6 mmol) was slowly added at 0 °C under nitrogen to a solution of TEG (10.0 g, 51.5 mmol) in anhydrous THF (40 mL). To this, methanesulfonyl chloride (15.1 mL, 195.6 mmol) was added over 30 min. under nitrogen, resulting in precipitation of a grey solid from a yellow solution. Stirring was continued at 0 °C for one more hour. After stirring for another 2 h at room temperature, the solution was chilled on ice/water bath and water (25 mL) added. The reaction was quenched with saturated sodium bicarbonate (15 mL) and the pH adjusted to 8 with sodium hydroxide (2.5 M). Approximately half the volume of THF was evaporated *in vacuo*, sodium azide (6.86 g, 105.6 mmol) added, and the reaction mixture refluxed overnight. The aqueous solution was extracted with diethyl ether (5 × 20 mL), the combined organic layers backwashed with brine (20 mL) and dried over anhydrous magnesium sulfate to yield a yellow oil. This oil was purified by flash chromatography over silica gel, with the column equilibrated in 12.5% ethyl acetate:hexane. A gradient of 12.5-50% ethyl acetate:hexane yielded TEG-diazide as a light yellow oil (8.7 g, 69%). ^{1}H-NMR (400 MHz, $CDCl_3$) δ 3.82-3.44 (m, $(H\text{-}3)_2$ – $(H\text{-}11)_2$), 3.37 (t, J = 8.0 Hz, $(H\text{-}2)_2$ and $(H\text{-}12)_2$). Mass spectrum (ESI+) *m/z*: 267 ($[M{+}Na]^+$), 262 ($[M{+}H_2O]^+$), 245

([M+H]$^+$), 239. ν_{max} (Neat film) 2872 (m), 2251 (s), 2108 (s), 1470 (m), 1346 (s), 1299 (m), 1124 (br), 907 (s), 743 (m), 649 (s).

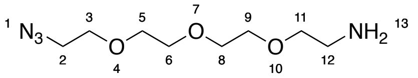

N_3-TEG-NH_2 (2-(2-(2-(2-azidoethoxy)ethoxy)ethoxy) ethanamine). TEG-diazide (1.0 g, 4.1 mmol) was dissolved in hydrochloric acid (1 M; 3 mL). With good stirring, triphenyl phosphine (1.18 g, 4.5 mmol) in ethylacetate (15 mL) was added slowly and stirred for a further 14 h. The organic layer was decanted and extracted with aqueous hydrochloric acid (1M; 2 × 3 mL). The combined aqueous layers were saturated with salt, made basic (to pH = 11-12) by addition of sodium hydroxide pellets and thereafter extracted with toluene (3 × 10 mL). The combined organic layers were washed with brine and dried over potassium hydroxide pellets. The solvents were removed *in vacuo*, leaving a yellow oily residue. This residue was purified by flash chromatography over silica gel, with the column equilibrated in dichloromethane. A gradient of 0-10% methanol:dichloromethane yielded N_3-TEG-NH_2 as a light yellow oil (5.5g, 61%). ^{1}H-NMR (400 MHz, $CDCl_3$) δ 3.66 – 3.56 (m, $(H\text{-}3)_2$ – $(H\text{-}11)_2$), 3.49 (t, J = 10.4 Hz, $(H\text{-}2)_2$), 3.35 (t, J = 8.0 Hz, $(H\text{-}12)_2$), 2.85 (b, $(H\text{-}13)_2$). Mass spectrum (ESI+) *m/z:* 241 ([M+Na]$^+$), 219 ([M+H]$^+$).

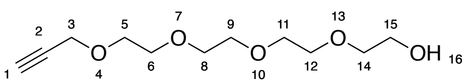

Acetylene-TEG-OH (3,6,9,12-tetraoxapentadec-14-yn-1-ol). To a solution of predried TEG (10.00 g, 51.5 mmol) in anhydrous THF (50 mL), sodium hydride (1.36 g, 56.6 mmol) was added slowly under nitrogen at 0 °C and stirred for 30 min. Freshly distilled propargyl bromide (4.59 mL, 51.5 mmol) was added at 0 °C over the next 30 min., kept on ice/water for another 2 h and stirred at room temperature overnight. The precipitate was filtered off, the solvent evaporated *in vacuo* and the yellow residue applied to flash chromatography over silica gel. The column was equilibrated to 99:1 dichloromethane:methanol. The residue was dissolved in the initial mobile phase and a gradient of 1-10% methanol:dichloromethane was developed yielding acetylene-TEG-OH as a light yellow oil (8.01 g, 67.1%). ^{1}H-NMR (400 MHz, $CDCl_3$) δ 4.11 (d, J = 2.4 Hz, $(H\text{-}3)_2$), 3.64 – 3.54 (m, $(H\text{-}5)_2$ – $(H\text{-}15)_2$), 2.38 (t, J = 3.6 Hz, H-1). Mass spectrum (ESI+) *m/z:* 254 ([M+Na]$^+$), 233 ([M+H]$^+$).

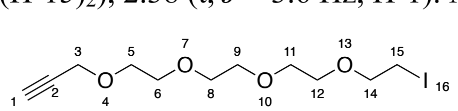

Acetylene-TEG-I (1-iodo-3,6,9,12-tetraoxapentadec-14-yne). A solution of alcohol (acetylene-TEG-OH; 200 mg, 0.86 mmol) in dry dichloromethane (1 mL) was added to a solution of iodine (120 mg, 0.95 mmol) and imidazole (64.5 mg, 0.95 mmol) in dry dichloromethane (1 mL) containing a suspension of polymer-bound triphenylphosphine (PPh_3) (320 mg resin equivalent to 0.95 mmol PPh_3) at 0 °C. The reaction mixture was allowed to warm to room temperature, while stirring was continued overnight. The polymer-bound PPh_3 was filtered off, the reaction mixture washed consecutively with an aqueous solution of saturated sodium thiosulfate, water and brine and then dried over anhydrous magnesium sulfate. The solvent was reduced to dryness *in vacuo*. A silica column was equilibrated in 7:3 hexane:ethyl acetate, developed with a gradient of 30-100% ethyl acetate:hexane yielding acetylene-TEG-I as a yellow oil (174 mg, 59%). ^{1}H-NMR (400 MHz, $CDCl_3$) δ 4.19 (d, J = 2.4 Hz, $(H\text{-}3)_2$), 3.55-3.71 (m, $(H\text{-}5)_2$ – $(H\text{-}14)_2$), 3.24 (t, J = 6.9 Hz, $(H\text{-}15)_2$), 2.41 (t, J = 2.4 Hz, H-1). ^{13}C-NMR (100 MHz, $CDCl_3$) δ 80.0 (C-2), 74.9 (C-1), 72.9-69.5 (C-5 – C-14), 62.1 (C-15), 58.8 (C-3). Mass spectrum (ESI+) *m/z:* 365 ([M+Na]$^+$), 360 ([M+H_2O]$^+$), 343 ([M+H]$^+$). (HRESI+) Found *m/z*: 365.0223, $C_{11}H_{19}IO_4Na$ requires 365.0220.

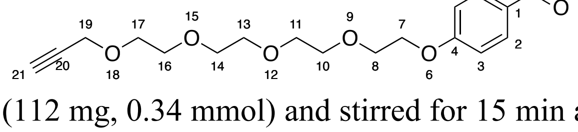

Acetylene-TEG-BA (4-(3,6,9,12-tetraoxapenta dec-14-ynyloxy)benzaldehyde). 4-Hydroxy-benzaldehyde (28 mg, 0.23 mmol) was dissolved in anhydrous acetonitrile (2 mL), added to cesium carbonate (112 mg, 0.34 mmol) and stirred for 15 min at room temperature. A solution of "acetylene-TEG-I" (117 mg, 0.34 mmol) in anhydrous MeCN (2 mL) was added and the stirring reaction mixture was reduced to 1 mL under nitrogen. After 72 h it was reduced to dryness under nitrogen. The residue was taken up in chloroform and loaded onto a silica column equilibrated in 99:1 chloroform:methanol. The column was developed with a gradient of 1-5% methanol:chloroform and 20 fractions were collected. Fractions 7-12

were combined (TLC) and reduced to dryness *in vacuo* yielding acetylene-TEG-BA as a light-yellow oil (74.2 mg, 96.4%). ^{1}H-NMR (400 MHz, $CDCl_3$) δ 9.86 (s, H-5), 7.81 (dt, *J* = 9.5 Hz, 2.4 Hz, (H-3)$_2$), 7.00 (dt, *J* = 9.5 Hz, 2.3 Hz, (H-2)$_2$), 4.22-4.15 (m, (H-7)$_2$ and (H-19)$_2$), 3.90-3.61 (m, (H-8 – H-17)$_2$), 2.41 (t, *J* = 2.4 Hz, H-21). ^{13}C-NMR (100 MHz, $CDCl_3$) δ 190.8 (C-5), 163.9 (C-1), 132.0 (C-3), 130.1 (C-2), 114.9 (C-4), 79.7 (C-20), 74.6 (C-21), 70.9-67.8 (8C, C-7 – C-17), 58.4 (C-19). Mass spectrum (ESI+) m/z: 359 ([M+Na]+), 337 ([M+H]+). (HRESI+) Found m/z: 359.1467, C18H24O6Na requires 359.1465.

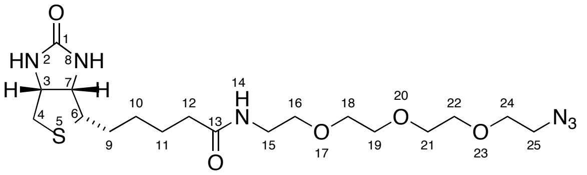

Biotin-TEG-N_3 (azidoethyl-di(ethylene glycol) ethylaminobiotin). A solution of NH_2-TEG-N_3 (70.3 mg, 0.32 mmol) in anhydrous acetonitrile (2.0 mL) was added to a solution of biotin *N*-hydroxysuccinimide ester (100 mg, 0.29 mmol) and TEA (82 µL, 0.59 mmol) in DMSO (0.2 mL). The reaction mixture was stirred for 18 h at 25 °C and reduced to dryness under a stream of nitrogen. The residue was taken up in chloroform and loaded onto a silica column equilibrated in 99:1 chloroform:methanol. The column was developed with a gradient of 1-20% methanol:chloroform and 60 fractions were collected. Fractions 44-57 were combined (TLC) and reduced to dryness *in vacuo* yielding Biotin-TEG-N_3 as a white sticky powder (102 mg, 78.5%). ^{1}H-NMR (400 MHz, DMSO-d_6) δ 7.81 (t, *J* = 5.4 Hz, H-14), 6.41 (s, H-8), 6.35 (s, H-2), 4.30 (m, H-3), 4.14 (m, H-7), 3.68 – 3.10 (m, (H-15)$_2$ – (H-25)$_2$), 3.20 (m, H-6), 2.82 (dd, *J* = 12.4 Hz, 5.1 Hz, H-4β), 2.57 (d, *J* = 12.6 Hz, H-4α), 2.06 (t, *J* = 7.4 Hz, (H-12)$_2$), 1.68 – 1.40 (m, (H-9)$_2$ – (H-11)$_2$). Mass spectrum (ESI+) *m/z*: 467 ([M+Na]$^+$), 445 ([M+H]$^+$).

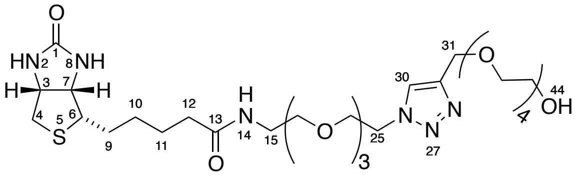

Biotin-TEG-triazole-TEG-OH. Copper(II) sulfate (0.1 mM; 0.25 mL, 23 µmol) was added to a solution of sodium ascorbate (9.1 mg, 46 µmol) in *tert*-butanol (0.25 mL) and stirred until the initially forming brown precipitate turned to a yellow solution (5 min). To this, a solution of Biotin-TEG-N_3 (250 mg, 0.56 mmol) and compound acetylene-TEG-OH (130 mg, 0.56 mmol) in *tert*-butanol:water (1:1; 1 mL) was added and stirred for 24 h. The reaction mixture was reduced under nitrogen and freeze-dried. The residue was suspended in 98:2 chloroform:methanol and loaded onto a silica column equilibrated in 99:1 chloroform:methanol. A gradient of 1-20% methanol:chloroform was applied yielding Biotin-TEG-triazole-TEG-OH as a light-yellow oil (290.3 mg, 76.1%). ^{1}H-NMR (400 MHz, DMSO-d_6) δ 8.03 (s, H-30), 7.80 (t, *J* = 5.4 Hz, H-14), 6.40 (s, H-8), 6.34 (s, H-2), 4.51 (s, (H-31)$_2$), 4.49 (t, *J* = 5.1 Hz, (H-25)$_2$), 4.29 (dd, *J* = 7.7 Hz, 5.0 Hz, H-3), 4.12 (m, H-7), 3.80 (t, *J* = 5.3 Hz, (H-24)$_2$), 3.63 – 3.26 (m, (H-16)$_2$ – (H-23)$_2$ and (H-33)$_2$ – (H-42)$_2$), 3.17 (m, (H-15)$_2$), 3.08 (m, H-6), 2.81 (dd, *J* = 12.7 Hz, 4.8 Hz, H-4β), 2.56 (d, *J* = 12.6 Hz, H-4α), 2.05 (t, *J* = 7.5 Hz, (H-12)$_2$), 1.60 (m, H-9a), 1.49 (m, (H-11)$_2$), 1.45 (m, H-9b), 1.31 (m, (H-10)$_2$). ^{13}C-NMR (100 MHz, $CDCl_3$) δ 174.6 (C-13), 162.7 (C-1), 143.8 (C-29), 124.2 (C-30), 72.3 – 69.5 (C-16 – C-24 and C-33 – C-42), 68.8 (C-24), 63.5 (C-31), 61.0 (C-7), 60.2 (C-43), 59.2 (C-3), 55.4 (C-6), 49.3 (C-25), 39.8 (C-4), 38.4 (C-15), 35.1 (C-12), 28.2 (C-10), 28.1 (C-9), 25.3 (C-11). Mass spectrum (ESI+) *m/z:* 700 ([M+Na]$^+$), 678 ([M+H]$^+$), 339 ([M+2H]$^{2+}$). (HRESI+) Found *m/z*: 699.3370, $C_{29}H_{52}N_6O_{10}S$ requires 699.3358.

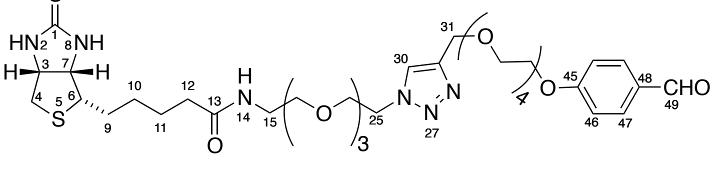

Biotin-TEG-triazole-TEG-BA. Copper(II) sulfate (0.1 mM; 0.25 mL, 23 µmol) was added to a solution of sodium ascorbate (9.1 mg, 46 µmol) in *tert*-butanol (0.25 mL) and stirred until the initially forming brown precipitate turned to a yellow solution (5 min). To this, a solution of Biotin-TEG-N_3 (34 mg, 76.5 µmol) and acetylene-TEG-BA (30.8 mg, 91.8 µmol) in *tert*-butanol:water (1:1, 1 mL) was added and stirred for 18 h. The reaction mixture was reduced under nitrogen and freeze-dried. The residue was suspended in 98:2 chloroform:methanol and loaded onto a silica column equilibrated in 99:1 chloroform:methanol. A

gradient of 1-20% chloroform:methanol was applied yielding the triazole as a light-brown oil (48.4 mg, 81.0%). ^{1}H-NMR (400 MHz, $CDCl_3$) δ 9.83 (s, H-49), 7.78 (dt, *J* = 9.5 Hz, 2.3 Hz, $(H\text{-}46)_2$), 7.72 (s, H-30), 6.98 (dt, *J* = 9.5 Hz, 2.2 Hz, $(H\text{-}47)_2$), 6.87 (t, *J* = 5.4 Hz, H-14), 6.43 (bs, H-8), 5.70 (bs, H-2), 4.62 (s, $(H\text{-}31)_2$), 4.49 (t, *J* = 5.1 Hz, $(H\text{-}25)_2$), 4.43 (m, H-3), 4.25 (m, H-7), 4.17 (t, *J* = 4.7 Hz, $(H\text{-}43)_2$), 3.83 – 3.46 (m, $(H\text{-}16)_2$ – $(H\text{-}24)_2$ and $(H\text{-}33)_2$ – $(H\text{-}42)_2$), 3.37 (m, $(H\text{-}15)_2$), 3.08 (m, H-6), 2.84 (dd, *J* = 12.7 Hz, 4.8 Hz, H-4β), 2.68 (d, *J* = 12.8 Hz, H-4α), 2.15 (t, *J* = 7.5 Hz, $(H\text{-}12)_2$), 1.73 – 1.33 (m, $(H\text{-}9)_2$ – $(H\text{-}11)_2$). ^{13}C-NMR (100 MHz, $CDCl_3$) δ 190.9 (C-49), 173.4 (C-13), 164.1 (C-48), 163.9 (C-1), 144.8 (C-29), 132.0 (C-46), 130.0 (C-45), 123.9 (C-30), 114.9 (C-47), 70.9 – 69.5 (C-16 – C-24 and C-33 – C42), 67.8 (C-43), 64.5 (C-31), 61.8 (C-7), 60.2 (C-3), 55.7 (C-6), 50.2 (C-25), 40.5 (C-4), 39.1 (C-15), 35.9 (C-12), 28.3 (C-10), 28.1 (C-9), 25.6 (C-11). Mass spectrum (ESI+) *m/z:* 803 ($[M+Na]^+$). (HRESI+) Found *m/z*: 803.3640, $C_{36}H_{56}N_6O_{11}SNa$ requires 803.3620.

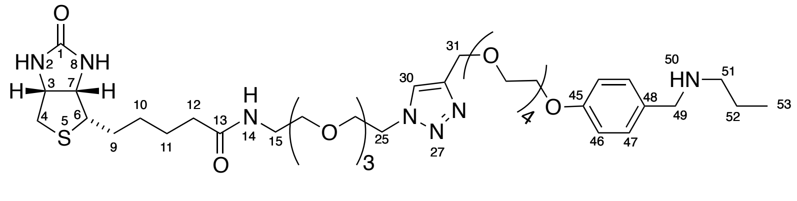

Biotin-TEG-triazole-TEG-BA-propylamine (B-PROP). To a solution of Biotin-TEG-triazole-TEG-BA (15.1 mg, 19.3 µmol) in methanol-d_4 (0.60 mL), was added $NaBH(OAc)_3$ (41.0 mg, 193.4 µmol) and 1-propylamine (9.5 µL, 116.0 µmol) and the reaction mixture stirred at ambient temperature for 5 min. ^{1}H-NMR showed the presence of imine [δ_H 8.16 (s, H-49)] and therefore more $NaBH(OAc)_3$ (25.0 mg, 118.0 µmol) was added. After another 0.5 h, NMR revealed the reaction was completed (no imine present) and the solvent was removed under a stream of nitrogen. The residue was suspended in chloroform and loaded onto a short silica column (5 cm) equilibrated in 95:5 chloroform:methanol. The column was developed with a gradient of 5-15% methanol:chloroform yielding B-PROP as a colourless oil (7.2 mg, 45%). ^{1}H-NMR (400 MHz, $CDCl_3$) δ 7.76 (s, H-30), 7.22 (d, *J* = 8.1 Hz, $(H\text{-}47)_2$), 6.85 (d, *J* = 8.5 Hz, $(H\text{-}46)_2$), 6.57 (t, *J* = 5.7 Hz, H-14), 6.02 (bs, H-8), 5.22 (bs, H-2), 4.67 (s, $(H\text{-}31)_2$), 4.53 (t, *J* = 3.4 Hz, $(H\text{-}25)_2$), 4.47 (m, H-3), 4.30 (m, H-7), 4.10 (t, *J* = 3.2 Hz, H-15), 3.87 (t, *J* = 3.35, H-43), 3.83 (t, *J* = 3.2 Hz, H-49), 3.79 – 3.36 (m, $(H\text{-}16)_2$ – $(H\text{-}24)_2$ and $(H\text{-}33)_2$ – $(H\text{-}42)_2$), 3.12 (m, H-6), 2.89 (dd, *J* = 12.8 Hz, 4.8 Hz, H-4β), 2.71 (d, *J* = 12.6 Hz, H-4α), 2.59 (t, *J* = 7.3 Hz, $(H\text{-}51)_2$), 2.18 (t, *J* = 7.0 Hz, $(H\text{-}12)_2$), 1.78 – 1.34 (m, $(H\text{-}9)_2$ – $(H\text{-}11)_2$), 1.53 (q, *J* = 7.3 Hz, H-52), 0.91 (t, *J* =7.4 Hz, $(H\text{-}53)_3$). ^{13}C-NMR (100 MHz, $CDCl_3$) δ 173.2 (C-13), 163.6 (C-1), 157.9 (C-45), 145.0 (C-29), 132.2 (C-48), 129.5 (C-47), 123.9 (C-30), 114.6 (C-46), 70.9 – 69.5 (C-16 – C-24 and C-33 – C-42), 67.5 (C-43), 64.6 (C-31), 61.8 (C-7), 60.1 (C-3), 55.5 (C-6), 53.2 (C-51), 51.0 (C-49), 50.2 (C-25), 40.6 (C-4), 39.2 (C-15), 35.9 (C-12), 28.2 (C-10), 28.1 (C-9) 25.5 (C-11), 22.9 (C-52), 11.8 (C-53). Mass spectrum (ESI+) *m/z:* 824 ($[M+H]^+$), 412 ($[M+2H]^+$). (HRESI+) Found *m/z*: 824.4588, $C_{39}H_{66}N_7O_{10}S$ requires 824.4586.

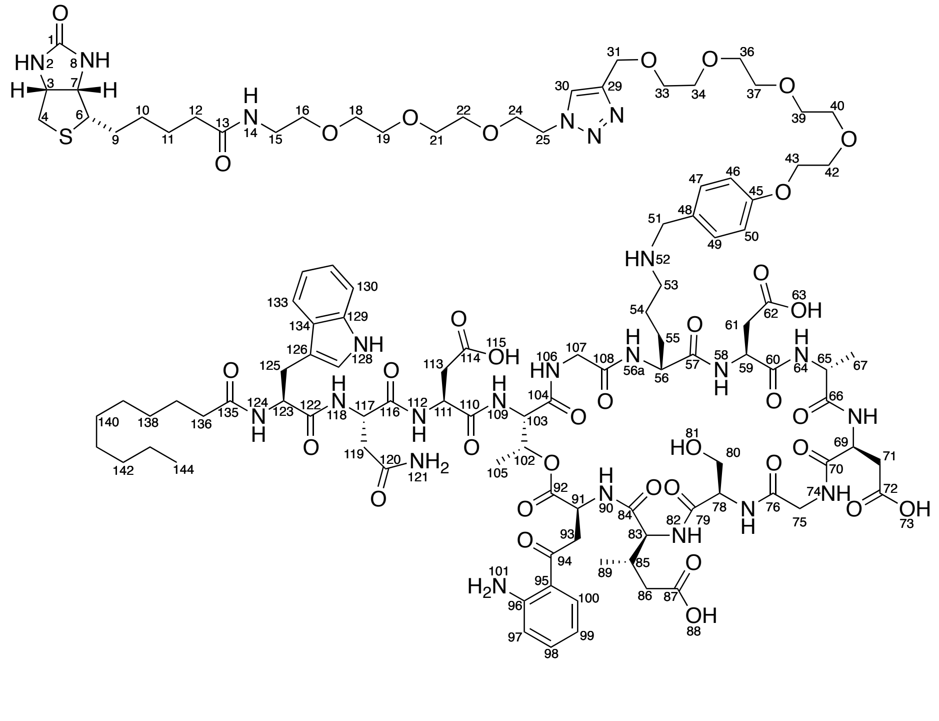

Biotin-daptomycin (B-DAP). A solution of Biotin-TEG-triazole-TEG-benzaldehyde (10.0 mg, 12.8 µmol) in dry DMF (200 µL) was added to daptomycin (20.7 mg, 12.8 µmol) under nitrogen and stirred for 1 h. To this, $NaBH(OAc)_3$ (16.3 mg, 76.8 µmol) was added and kept stirring at ambient temperature for 24 h. The solvent was removed under a stream of nitrogen. The residue was dissolved in 5:95 methanol:water (1 mL) and separated by HPLC

(analytical Vydac C4 250.0 × 4.60, 5μ, 300Å, 1 mL/min, gradient from 7-30% acetonitrile:5mM ammonium phosphate buffer pH = 3.2, over 30 min). The peak eluting after 20.1 min was collected and freeze-dried. The residue was taken up in 5:95 methanol:water (2 mL) and loaded onto a disposable C18 cartridge (200 mg bed weight). The cartridge was washed with water (3 mL) to remove any salts and the compound eluted with methanol. The solvent was evaporated *in vacuo* yielding B-DAP as light yellow solid (8.4 mg, 27%). ^{1}H-NMR and selected 2D-NMR spectra are displayed in Figures S1 and S2. Mass spectrum (ESI+) m/z: 1193.6 ($[M+2H]^{2+}$), 796.1 ($[M+3H]^{3+}$). (HRESI+) Found m/z: 1215.0312. $C_{108}H_{157}N_{23}O_{36}SNa_2$ requires 1215.0333.

2D-NMR data (HSQC, HMBC, COSY, ROESY and NOESY) acquired for B-DAP showed that it was approximately a 1:2 mixture of B-DAP and of an oxidized analogue incorporating biotin sulfoxide (B_{Ox}-DAP). This is manifested as two subsets of urea N$\underline{H}$ signals (δ_H 6.78 / 6.69 and 6.42 / 6.37) in the HMBC and COSY data (Figure S2) [2]. MS studies (below) provided further evidence for the presence of B-DAP and B_{Ox}-DAP.

In MS/MS studies, first a full scan between 400-2000 amu revealed the doubly charged proton and potassium ion of B-DAP ($[M_1+H+K]^{2+}$) at 1212.3 amu (Figure S3, panel A) as the most abundant signal. The biotin-sulfoxide probe [M_2] was identified as di-protonated species ($[M_2+2H]^+$) at 1201.3 amu, and furthermore as the potassium and sodium adducts ($[M_2+K+H]^{2+}$ or $[M_2+Na+H]^{2+}$). The signal for ($[M_1+H+K]^{2+}$) was isolated for MS-MS (Figure S3, panel C).

Fragmentation was performed via alternating collision induced detection (CID) and electron transfer detection (ETD). In MS^2 of the above parent ion, the major two fragments were identified as the biotinylated linker including the amine group of ornithine and the potassium salt of daptomycin lacking the ornithine amino group (Figure S3, panel C). MS^3 studies of the daptomycin fragment revealed two primary fragmentation pathways of nor-daptomycin (Figures S4 and S5), i.e. pathways 2A and 2B. The starting point for both pathways was the potassium adduct of nor-daptomycin. Pathway 2A started with a ring opening between Nor6 and Asp7 and included an immediate loss of water. In parallel, pathway 2B was observed, which showed a ring-opening of nor-daptomycin at the lactone between Thr4 and Kyn13. Further fragmentation patterns matched the amino acid sequences for the ring opened initial fragment. Due to the initial loss of the TEG-biotin with an amino group and the consequent fragmentation pattern showing that the rest of the molecule was intact, it was concluded that reductive amination had occurred on the ornithine side chain as outlined in Figure S4.

For simplicity reasons, the mixture of B-DAP and B_{Ox}-DAP are further referred to as B-DAP.

Purity test of B-DAP. The purity of B-DAP was verified by reinjecting an aliquot (30 μL; 0.21 μM in 20% acetonitrile:5 mM ammonium phosphate buffer, pH =3.2) onto an analytical C18 RP-HPLC column (1 mL/min, 20–70% acetonitrile:5mM ammonium phosphate buffer over 30 min). The trace contained exclusively one peak, which eluted after 21.8 min and showed absorbance maxima at 224 nm, 259 nm and 369 nm (Figure S6, top panel).

Stability test of B-DAP. A solution of B-DAP in PBS was incubated at room temperature and aliquots analyzed by RP-HPLC over 12 h on an analytical C18 RP-HPLC column (1 mL/min, 25–80% acetonitrile:5 mM ammonium phosphate buffer (pH =3.2) over 40 min; Figure S6, bottom panel). Integration (Figure S6, insert) indicated that hydrolysis of B-DAP occurs very slowly (1% over 12 h), and hence B-DAP was regarded stable enough for the biopanning experiments (~ 6 h). PBS was used as a negative control.

Antibiotic activity of B-DAP. Biosafety approval was obtained from the Macquarie University Biosafety Committee (approval number 5201000870). The strains of bacteria used was *Staphylococcus aureus* ATCC 9144 (obtained from the CDS Reference Laboratory, Department of Microbiology, The Prince of Wales Hospital, NSW, Australia). Glycerol stock cultures (20% v/v glycerol) of the strain were kept at –80 °C. Initial cultures were prepared by streaking a small quantity of the frozen stock onto an LB agar plate and incubation at 37 °C for 16 h. The plates were stored at 4°C and used to inoculate subsequent cultures for up to one month. Overnight cultures were generally prepared by inoculating the according medium with a single bacterial colony from the agar plates, and subsequent incubation at 37

°C for 16 h with shaking (100–150 rpm). The optical density at 600 nm (OD_{600}) was adjusted to 0.08–0.1 to produce an inoculum density of 1.0×10^8 cfu/mL (OD).

For the turbidity microdilution assay, the bacterial strain was grown overnight in Mueller Hinton broth (MHB) (15 mL). The assay was performed in sterile, clear flat-bottom-96-well microtiter plates. The arrangement of samples and controls was outlined according to Appendino [3]. DAP, B-DAP or ampicillin (5.0 mg) were dissolved in DMSO (1 mL) and the final volume was made up to 5.0 mL with distilled water. Using a 96 well microtiter plate, MHB was dispensed into wells 1–11 (125 µL each) for each row, the antibiotic solution was added to well 1 (125 µL; in different rows for each antibiotic/replicate) and mixed thoroughly, after which 125 µL was taken out and dispensed to the next well (i.e. well 2). This process of two-fold serial dilution was carried out until well 10, and skipping well 11, the final volume was dispensed into well 12. Again, 125 µL each of the bacterial inoculum was dispensed into wells 1 to 11 leaving well 12 free of inoculum. Well 11 was free of the test or control compound, thus this acted as a positive growth control. Similarly, well 12 served as sterile control of the assay. 5% DMSO was also included as a negative control. The optical density at 600 nm (OD_{600}) was measured and the plate was incubated at 37 °C for 18 h. After incubation, the OD_{600} was determined again and the pre-incubation values subtracted. The resulting optical densities were converted to %(growth), plotted against molar concentration of the compounds and analyzed in GraphPad Prism to yield LD_{50} values (Fig. S8).

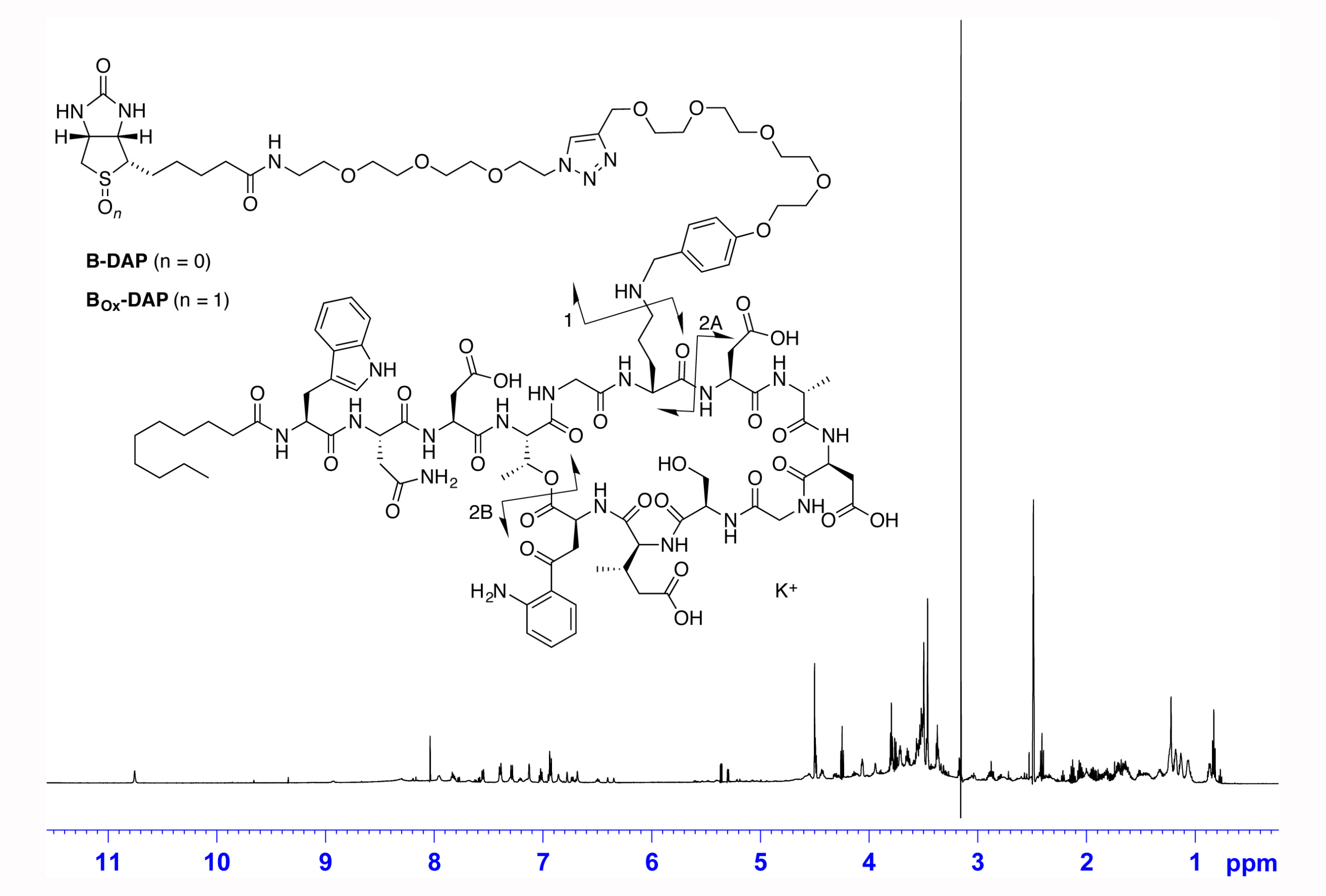

Figure S1. ^{1}H NMR spectra and structure of 2:1 mixture of B-DAP and B_{Ox}-DAP (DMSO-d_6, 600 MHz)

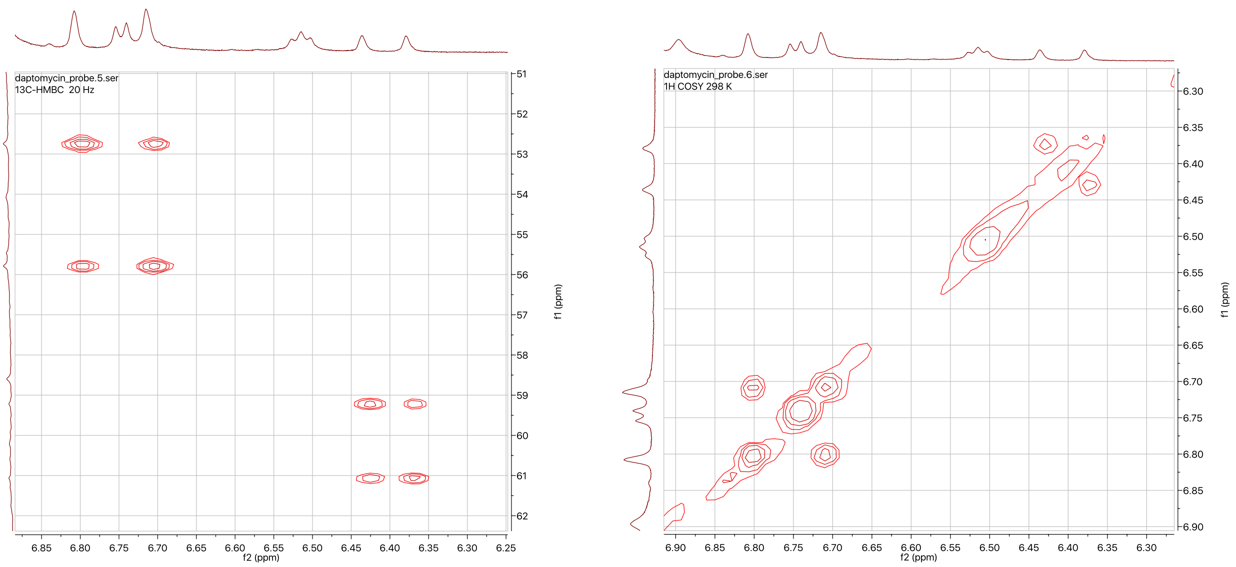

Figure S2: Selected HMBC (left panel) and COSY (right panel) couplings (DMSO-d_6, 600 MHz) of the biotin urea N-H protons: two sets of the long range couplings for B-DAP (δ_H 6.42, 6.37) and B_{Ox}-DAP (δ_H 6.78, 6.69), present in approximately a 2:1 ratio.

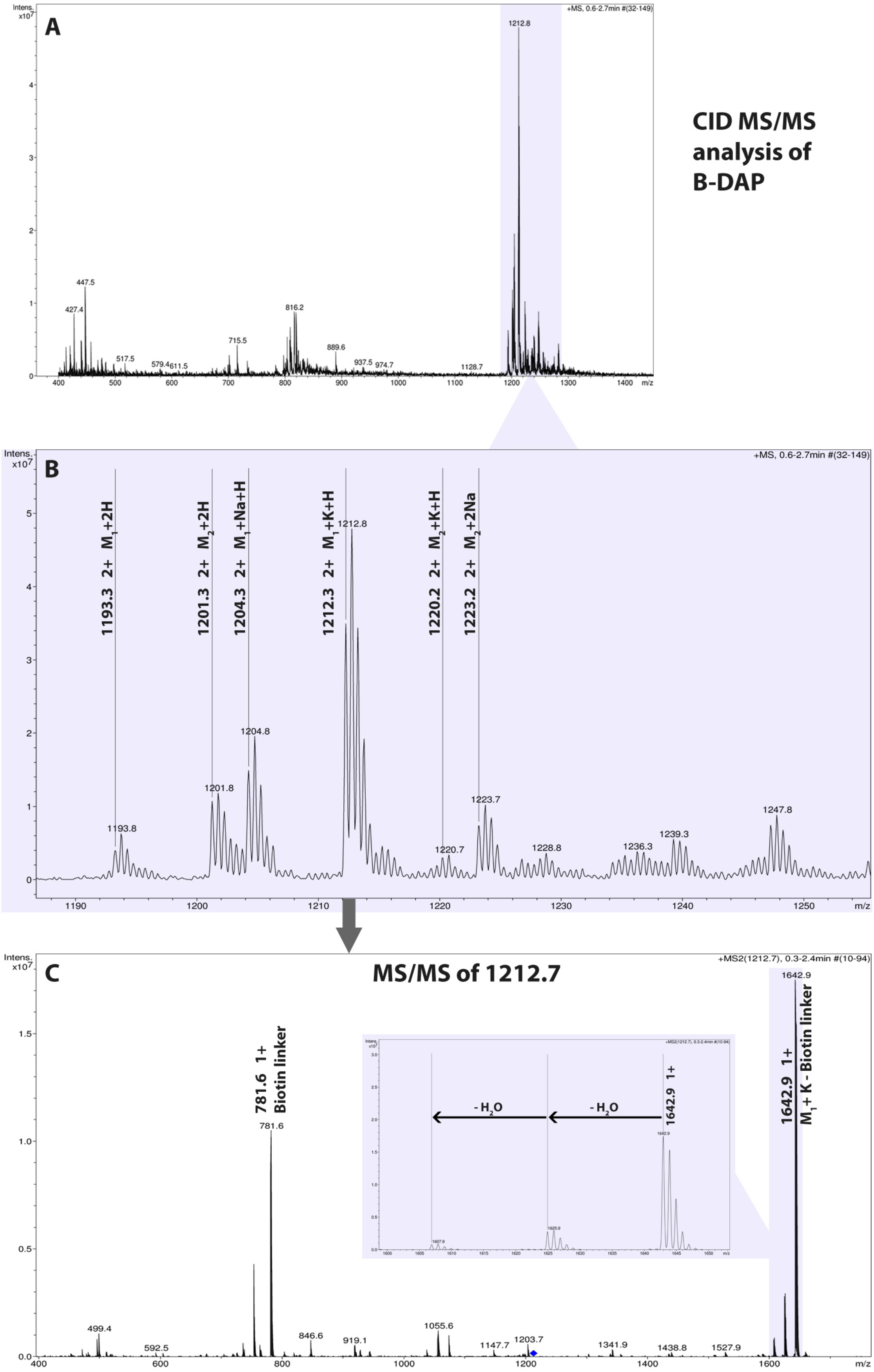

Figure S3: MS spectrum and MS^2 data of B-DAP: (A) MS spectrum of scan between 400-1500 amu; (B) Expanded MS chromatogram between 1185-1255 amu and identified adduct ions of B-DAP [M_1] and B_{Ox}-DAP [M_2]; (C) MS^2 of B-DAP (1212.7 amu) showing two main fragments - the biotinylated linker and K^+-adduct of Nor-daptomycin (missing the ornithine amine).

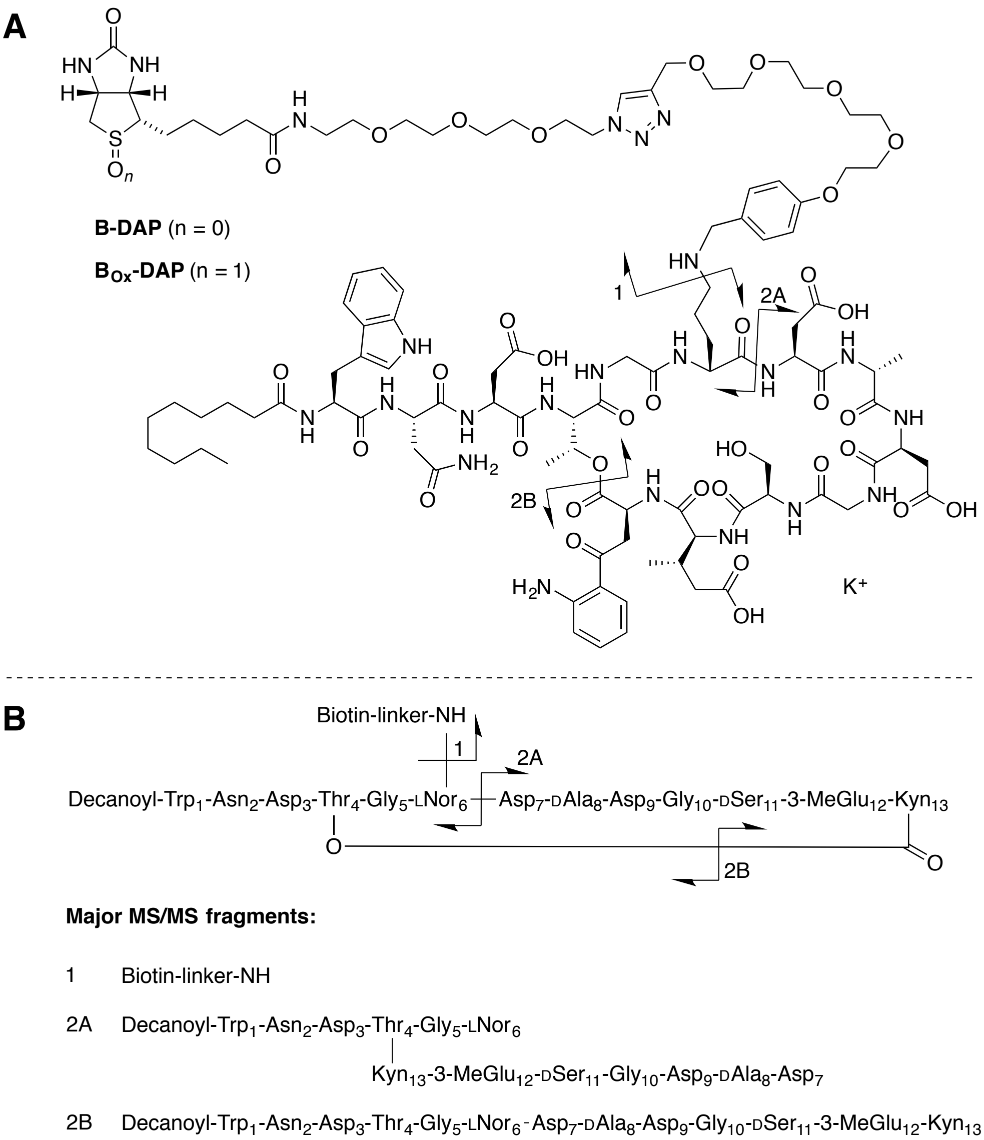

Figure S4: (A) Structure of B-DAP and B$_{Ox}$-DAP. (B) Schematic representation of the amino acid sequence of B-DAP including sites of fragmentation (1, 2A and 2B) during direct injection ESI-MS/MS analysis and schematic representation of major MS/MS fragments.

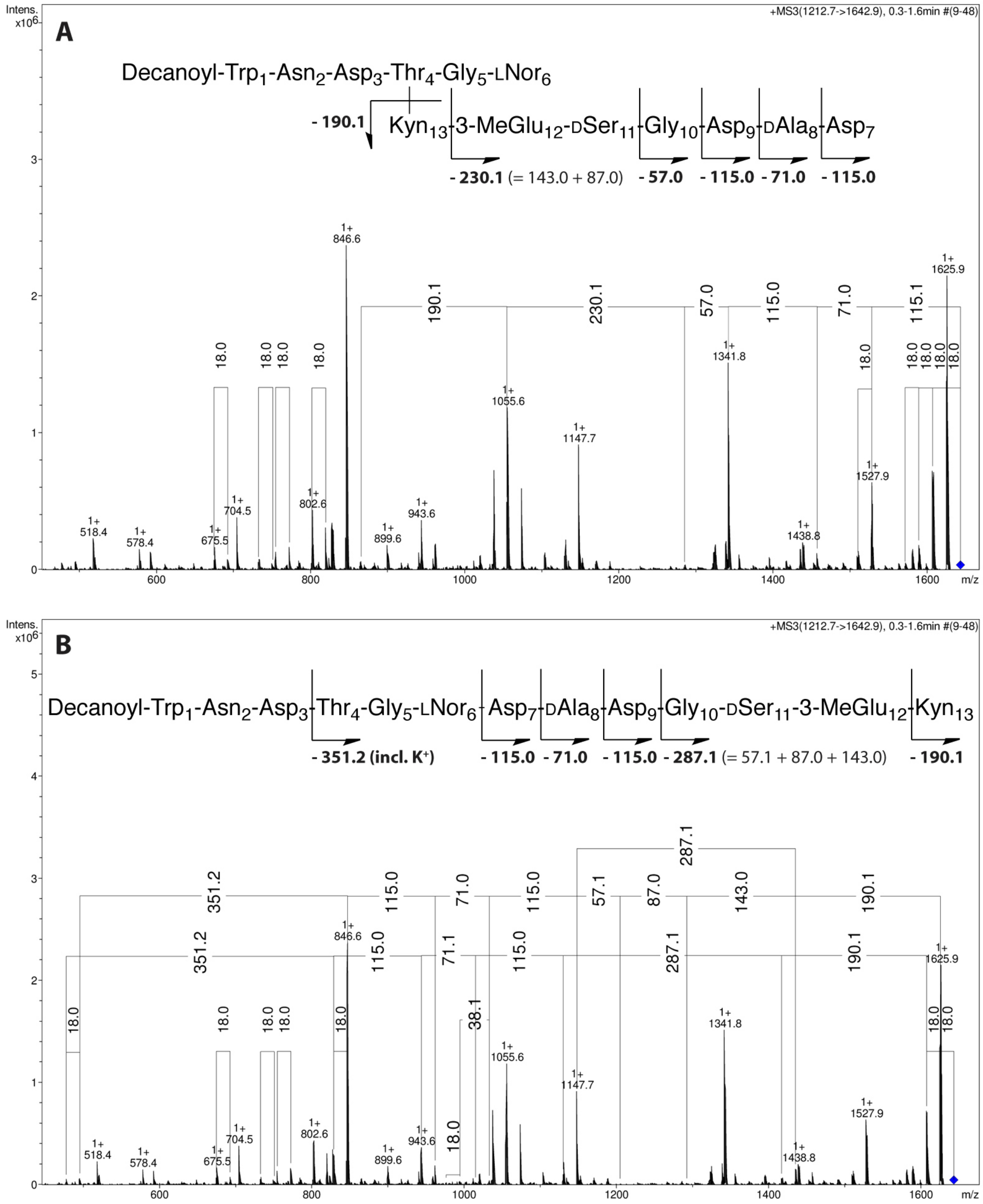

Figure S5: MS^3 data of Nor-daptomycin, showing fragmentation patterns of fragments 2A and 2B (Figure S4). (A, fragment 2A) Nor-daptomycin ring-opens between L-Nor6 and Asp7 into a bifurcated species and undergoes sequential loss of amino acids at position 7-12. (B, fragment 2B). A ring-opened, linear nor Daptomycin variant shows MS^3 fragments matching a sequential loss of single amino acids or peptides from positions 13 to 3.

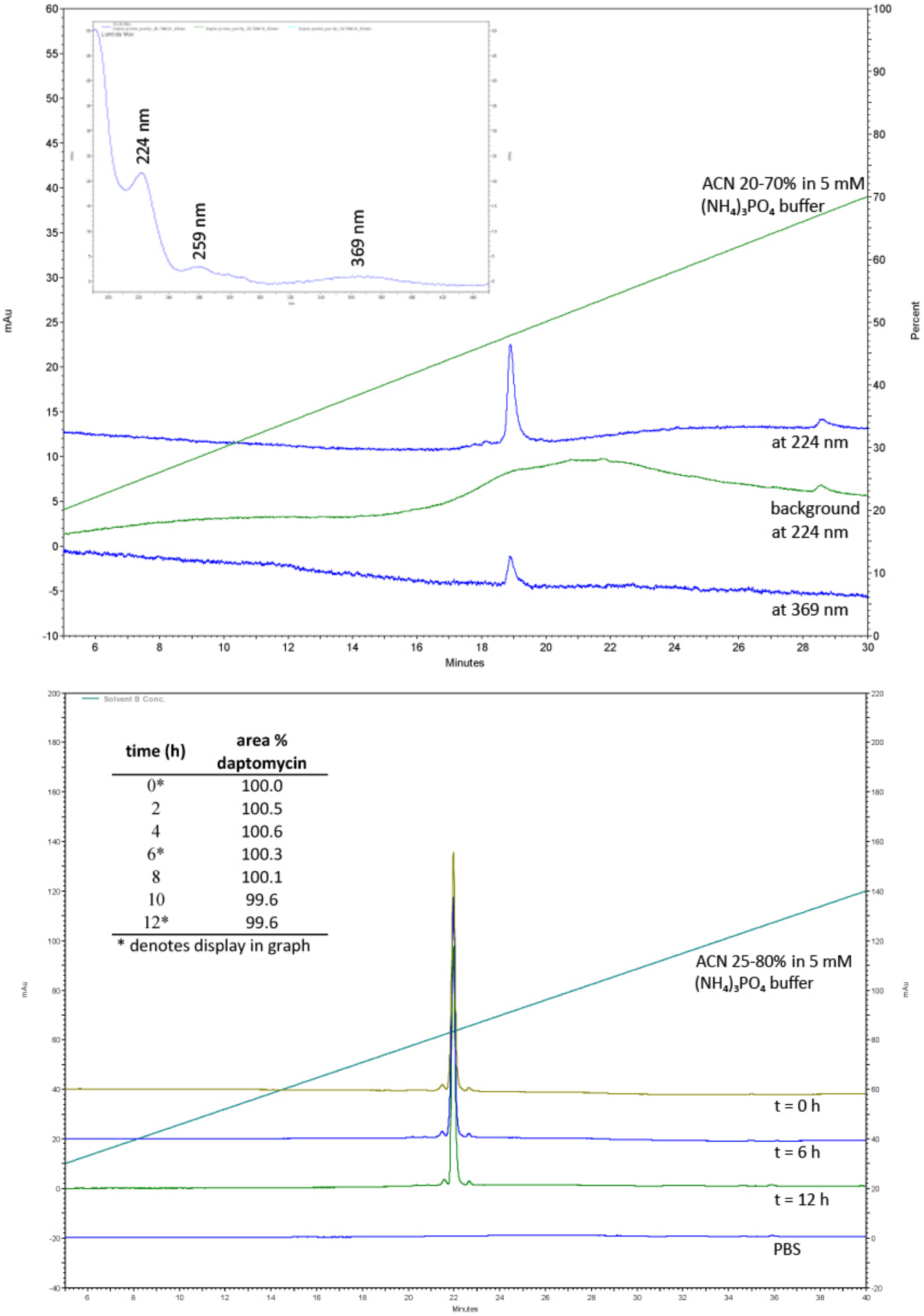

Figure S6: Purity and stability analysis of B-DAP in solution (30 µL; 0.21 µM) (a, top panel) HPLC UV traces (224 nm and 365 nm) and UV spectrum of purified B-DAP in 25% acetonitrile:5mM ammonium phosphate buffer on an analytical C18 RP-HPLC column (1 mL/min, 20–70% acetonitrile:5mM ammonium phosphate buffer pH =3.2 over 30 min). The trace contained exclusively one peak, which eluted after 18.8 min and showed absorbance maxima at 224 nm, 259 nm and 369 nm. (b, bottom panel) HPLC UV traces (at 230 nm) after treating B-DAP in PBS for 0, 6, and 12 h in comparison to background (PBS), analyzed an analytical C18 RP-HPLC column (1 mL/min, 25–80% acetonitrile:5mM ammonium phosphate buffer pH = 3.2 over 40 min).

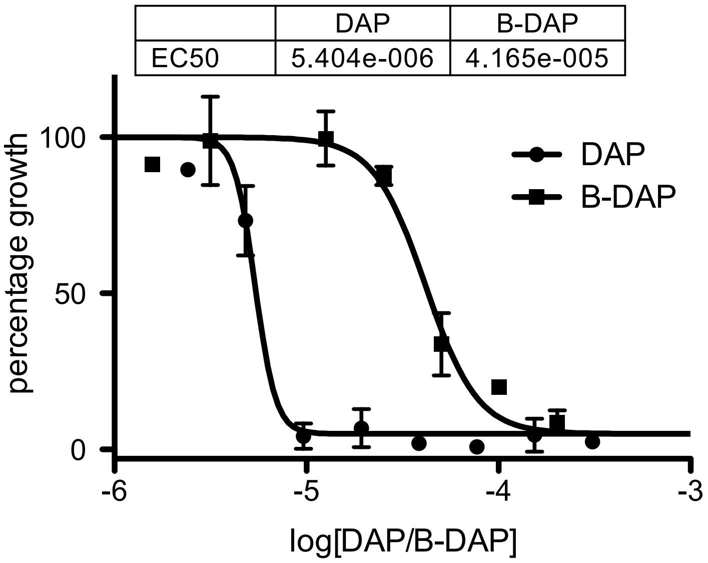

Figure S7: Antibacterial assay of DAP and B-DAP on *S. aureus*, displayed as percent growth versus concentration in mol/L. Data was fitted to a sigmoidal dose-response curve with variable slope (Hill plot) with the top and bottom values set to 100 and 0, respectively.

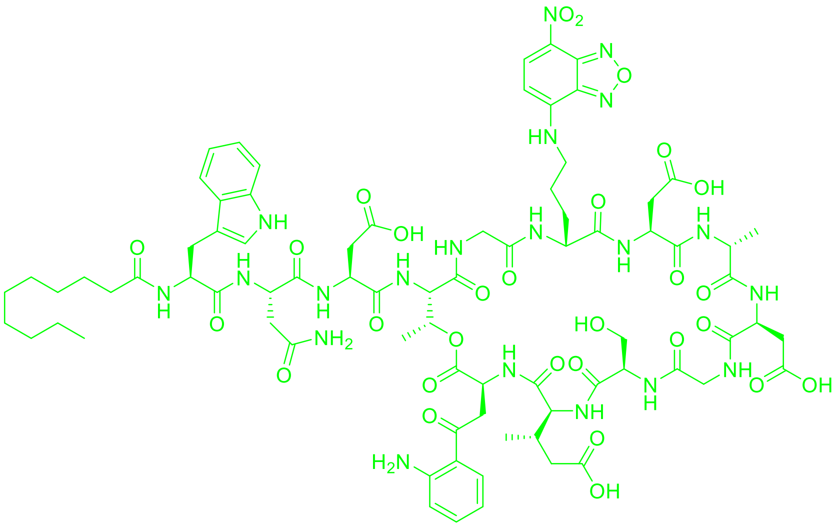

Daptomycin-NBD (F-DAP).

F-DAP was synthesized by the method of Muraih et al. [4]. Briefly, to a solution of daptomycin (10 mg, 6.17 µmol) in 50 mM sodium borate buffer (pH 8), containing 20 mM EDTA (10 mL), was added a solution of NBD-Cl (12.3 mg, 61.7 µmol) in acetonitrile (3 mL), and the mixture was incubated at 60 °C for 2 h. The mixture was then cooled in an ice water bath for 2 min, and finally acidified by adding 13 mL of acetic acid (50 mM). The reaction mixture was freeze-dried and the residue triturated with methanol (2 × 2 mL). and the combined extracts diluted with water (2 mL). The solution was then separated by HPLC (GIS-C18 column, 150 × 10.0 mm i.d., 5 µm, 5 mL min^{-1}, 5 mL min^{-1}, gradient from 35-50% acetonitrile (+0.05% TFA) in aqueous TFA (0.05%) over 30 min. The peak eluting at 23.5 min was collected and freeze-dried to yield pure F-DAP as a yellow powder (3.0 mg, 27 %). Mass spectrum (ESI+) m/z: 892.4 ([M+2H]$^{2+}$), 595.5 ([M+3H]$^{3+}$).

Table S1. Preparation of reagents and media used in display cloning

Reagent	Ingredients	Instructions
PBS	0.01 M KH_2PO_4<br>0.138 M NaCl<br>0.0027 M KCl	- Adjust pH to 7.4<br>- Autoclave 20 min / 121 °C
PWB	0.01 M KH_2PO_4<br>0.138 M NaCl<br>0.0027 M KCl<br>0.05% Tween-20	- Adjust pH to 7.4<br>- Autoclave 20 min / 121 °C
20xM9	20 g NH_4Cl<br>60 g KH_2PO_4<br>120 g $Na_2HPO_4.12H_2O$<br>Water to make 1 L of solution	- Adjust pH to 7.4<br>- Autoclave 20 min / 121 °C
20% Glucose	200 g glucose<br>Water to make 1 L of solution	- Autoclave 20 min / 121 °C
5%Carbenicillin	1 g carbenicillin disodium<br>Water to make 20 mL of solution	- Sterile filter (0.22 μm)<br>- Store at -20 °C
50xTAE	242 g Tris<br>57.1 mL acetic acid<br>18.6 g EDTA disodium salt<br>Water to make 1 L of solution	- Adjust pH to 8.0<br>- Autoclave 20 min / 121 °C
M9TB	9.28 g tryptone<br>4.64 g NaCl<br>50 mL 20x M9<br>1 mL $MgCl_2$ (1M)<br>Water to make 1 L of solution	- Autoclave 20 min / 121 °C<br>- Cool to room temperature<br>- Add 20 mL 20% glucose<br>- Add 1 mL 5% carbenicillin
2xYT	17 g tryptone<br>10 g yeast extract<br>5 g NaCl<br>Water to make 1 L of solution	- Adjust pH to 7.4<br>- Autoclave 20 min / 121 °C
LB Agar	10 g tryptone<br>5 g yeast extract<br>10 g NaCl<br>15 g agar<br>Water to make 1 L of solution	- Adjust pH to 7.4<br>- Autoclave 20 min / 121 °C<br>- Cool to 55 °C<br>- Pour plates (~100)<br>- Store plates at 4 °C
LB Agarose	10 g tryptone<br>5.2 g yeast extract<br>5.2 g NaCl<br>6 g agarose<br>Water to make 1 L of solution	- Adjust pH to 7.4<br>- Autoclave 20 min / 121 °C<br>- Cool to 55 °C<br>- Store in 25 mL lots at 4 °C
PCR Master Mix	10 μL 'UP' Primer (10 μM)<br>10 μL 'DOWN' Primer (10 μM)<br>20 μL dNTPs (10 mM each dNTP)<br>100 μL Taq buffer (10x)<br>835 μL nuclease free water	- Store at –20 °C<br><br>- Defrost and add 5 μL Taq Polymerase before use

Table S2. Standard thermocycler program for PCR of cDNA inserts

Cycles	Temperature	Time
1	94 °C	150 s
20	94 °C<br>55 °C<br>72 °C	45 s<br>60 s<br>30 s

1	72 °C	10 min
1	4 °C	∞

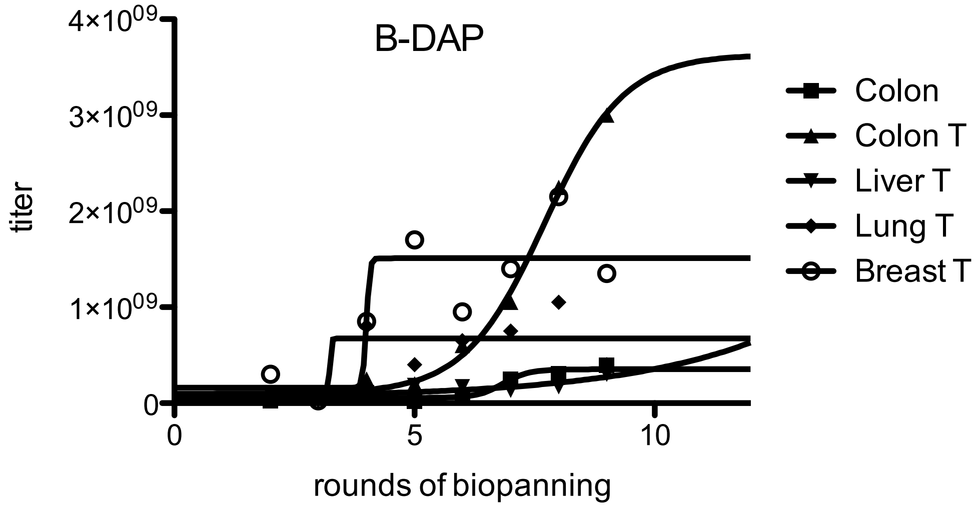

Figure S8. Phage titer (pfu/mL) after biopanning of B-DAP against five cDNA libraries (normal colon, colon tumor, breast tumor, liver tumor, lung tumor), showing exponential increase of titer over 12 or 9 rounds of selection for colon or others, respectively. Titers are fitted to sigmoidal dose-response curve (variable slope)

Table S3. Sequence alignment of RPS19 clones isolated from colon tumor (CoT), liver tumor (LiT), lung tumor (LuT) and breast tumor (BrT) cDNA phage libraries and consensus (red) and compared with human RPS19 (NCBI ID: NP_001013.1; blue). Each library converged on many different clones with varying amounts of 3'-UTR. All RPS19 cDNA inserts were in-frame with the coat protein.

```
                      10         20         30         40         50
              ....|....| ....|....| ....|....| ....|....| ....|....|
CoT_A4     :  ---------- ---------- -SKKSGKLKV PEWVDTVKLA KHKELAPYDE
LiT_C1     :  ---------- ---------- ---------- --SVDTVKLA KHKELAPYDE
LiT_E1     :  ---------- ---------- ---------- --------SA KHKELAPYDE
LuT_C4     :  ---------- ---------- ---------- -----SVKLA KHKELAPYDE
BrT_D7     :  ---------- ---------- ---------- -----GVKLA KHKELAPYDE
BrT_A9     :  ---------- ---------- ---------- --SVDTVKLA KHKELAPYDE
RPS19      :  -PGVTVKDVN QQEFVRALAA FLKKSGKLKV PEWVDTVKLA KHKELAPYDE
Consensus  :  ---------- ---------- ---------- ---------A KHKELAPYDE

                      60         70         80         90        100
              ....|....| ....|....| ....|....| ....|....| ....|....|
CoT_A4     :  NWFYTRAAST ARHLYLRGGA GVGSMTKIYG GRQRNGVMPS HFSRGSKSVA
LiT_C1     :  NWFYTRAAST ARHLYLRGGA GVGSMTKIYG GRQRNGVMPS HFSRGSKSVA
LiT_E1     :  NWFYTRAAST ARHLYLRGGA GVGSMTKIYG GRQRNGVMPS HFSRGSKSVA
LuT_C4     :  NWFYTRAAST ARHLYLRGGA GVGSMTKIYG GRQRNGVMPS HFSRGSKSVA
BrT_D7     :  NWFYTRAAST ARHLYLRGGA GVGSMTKIYG GRQRNGVMPS HFSRGSKSVA
BrT_A9     :  NWFYTRAAST ARHLYLRGGA GVGSMTKIYG GRQRNGVMPS HFSRGSKSVA
RPS19      :  NWFYTRAAST ARHLYLRGGA GVGSMTKIYG GRQRNGVMPS HFSRGSKSVA
Consensus  :  NWFYTRAAST ARHLYLRGGA GVGSMTKIYG GRQRNGVMPS HFSRGSKSVA

                     110        120        130        140
              ....|....| ....|....| ....|....| ....|....| ....|
CoT_A4     :  RRVLQALEGL KMVEKDQDGG RKLTPQGQRD LDRIAGQVAA ANKKH
LiT_C1     :  RRVLQALEGL KMVEKDQDGG RKLTPQGQRD LDRIAGQVAA ANKKH
LiT_E1     :  RRVLQALEGL KMVEKDQDGG RKLTPQGQRD LDRIAGQVAA ANKKH
LuT_C4     :  RRVLQALEGL KMVEKDQDGG RKLTPQGQRD LDRIAGQVAA ANKKH
BrT_D7     :  RRVLQALEGL KMVEKDQDGG RKLTPQGQRD LDRIAGQVAA ANKKH
BrT_A9     :  RRVLQALEGL KMVEKDQDGG RKLTPQGQRD LDRIAGQVAA ANKKH
RPS19      :  RRVLQALEGL KMVEKDQDGG RKLTPQGQRD LDRIAGQVAA ANKKH
Consensus  :  RRVLQALEGL KMVEKDQDGG RKLTPQGQRD LDRIAGQVAA ANKKH
```

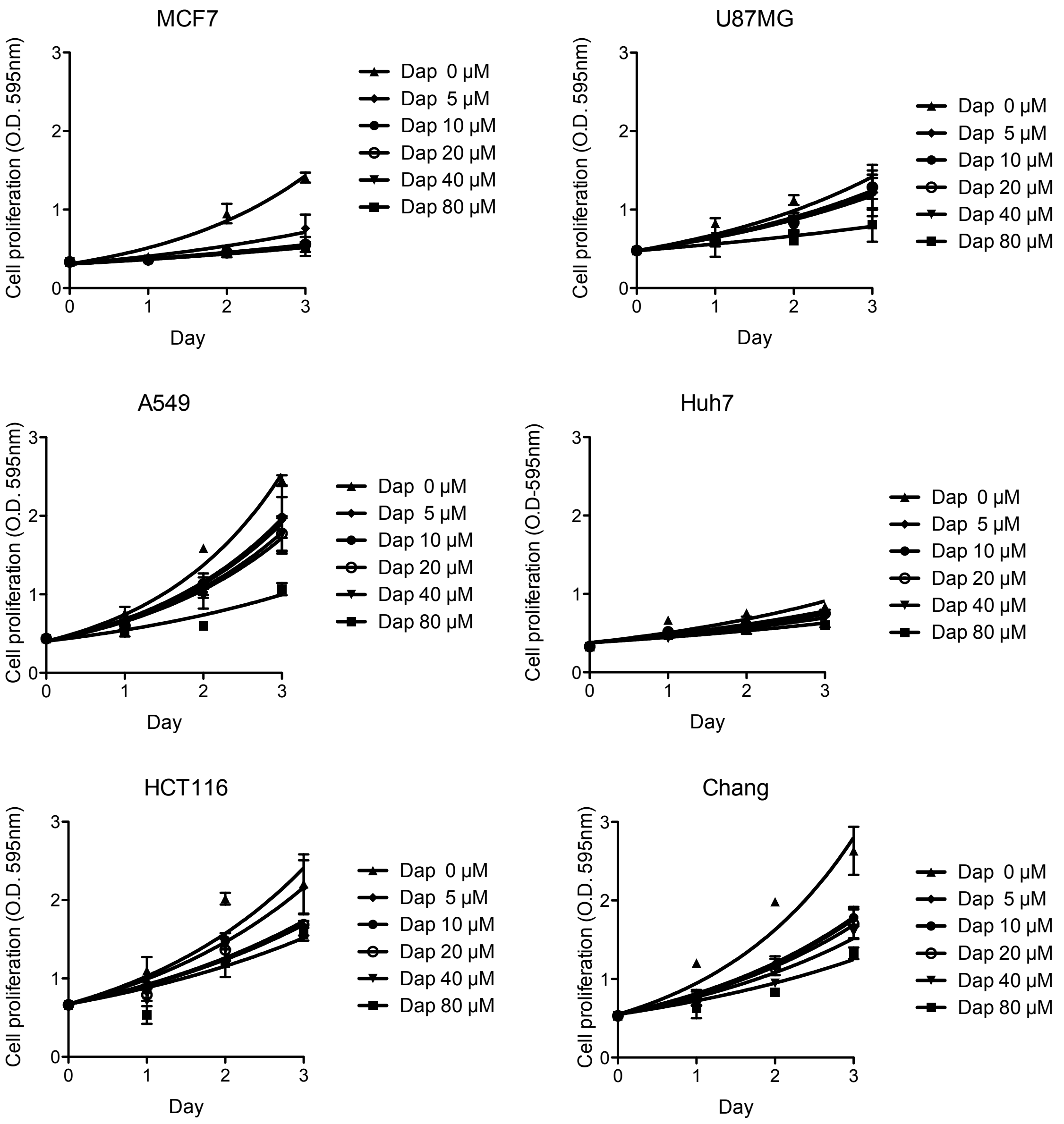

Figure S9. The effect of DAP on the growth of various cell lines.

2. References

1. Spura A, Riel RU, Freedman ND, Agrawal S, Seto C, Hawrot E: **Biotinylation of Substituted Cysteines in the Nicotinic Acetylcholine Receptor Reveals Distinct Binding Modes for Î±-Bungarotoxin and Erabutoxin a.** *J Biol Chem* 2000, **275:**22452-22460.
2. Liu F-T, Leonard NJ: **Avidin-biotin interaction. Synthesis, oxidation, and spectroscopic properties of linked models.** *J Am Chem Soc* 1979, **101:**996-1005.
3. Appendino G, Gibbons S, Giana A, Pagani A, Grassi G, Stavri M, Smith E, Rahman M: **Antibacterial Cannabinoids from Cannabis sativa: A Structure-Activity Study.** *J Nat Prod* 2008, **71:**1427-1430.
4. Muraih JK, Pearson A, Silverman J, Palmer M: **Oligomerization of daptomycin on membranes.** *Biochimica et Biophysica Acta - Biomembranes* 2011, **1808:**1154-1160.
